# Efficacy and safety of a four-drug, quarter-dose treatment for hypertension: the QUARTET USA randomized trial

**DOI:** 10.1038/s41440-024-01658-y

**Published:** 2024-04-08

**Authors:** Mark D. Huffman, Abigail S. Baldridge, Danielle Lazar, Hiba Abbas, Jairo Mejia, Fallon M. Flowers, Adriana Quintana, Alema Jackson, Namratha R. Kandula, Donald M. Lloyd-Jones, Stephen D. Persell, Sadiya S. Khan, James J. Paparello, Aashima Chopra, Priya Tripathi, My H. Vu, Clara K. Chow, Jody D. Ciolino

**Affiliations:** 1 Cardiovascular Division and Global Health Center, Washington University in St. Louis, St. Louis, MO USA; 2 The George Institute for Global Health, University of New South Wales, Sydney, NSW Australia; 3 Department of Preventive Medicine, Feinberg School of Medicine, Northwestern University, Chicago, IL USA; 4 Bluhm Cardiovascular Institute, Northwestern Medicine, Chicago, IL USA; 5 Access Community Health Network, Chicago, IL USA; 6 Division of General Internal Medicine, Department of Medicine, Feinberg School of Medicine, Northwestern University, Chicago, IL USA; 7 Division of Cardiology, Department of Medicine, Feinberg School of Medicine, Northwestern University, Chicago, IL USA; 8 Center for Primary Care Innovation, Institute for Public Health and Medicine, Feinberg School of Medicine, Northwestern University, Chicago, IL USA; 9 Division of Nephrology and Hypertension, Department of Medicine, Feinberg School of Medicine, Northwestern University, Chicago, IL USA; 10 Westmead Applied Research Centre, University of Sydney, Westmead, NSW Australia; 11 Northwestern University Data Analysis and Coordinating Center, Feinberg School of Medicine, Northwestern University, Chicago, IL USA

**Keywords:** Hypertension, Single pill combination, Quadpill, Randomized trial, Federally qualified health centers

## Abstract

New approaches are needed to lower blood pressure (BP) given persistently low control rates. QUARTET USA sought to evaluate the effect of four-drug, quarter-dose BP lowering combination in patients with hypertension. QUARTET USA was a randomized (1:1), double-blinded trial conducted in federally qualified health centers among adults with hypertension. Participants received either a quadpill of candesartan 2 mg, amlodipine 1.25 mg, indapamide 0.625 mg, and bisoprolol 2.5 mg or candesartan 8 mg for 12 weeks. If BP was >130/>80 mm Hg at 6 weeks in either arm, then participants received open label add-on amlodipine 5 mg. The primary outcome was mean change in systolic blood pressure (SBP) at 12 weeks, controlling for baseline BP. Secondary outcomes included mean change in diastolic blood pressure (DBP), and safety included serious adverse events, relevant adverse drug effects, and electrolyte abnormalities. Among 62 participants randomized between August 2019-May 2022 (*n* = 32 intervention, *n* = 30 control), mean (SD) age was 52 (11.5) years, 45% were female, 73% identified as Hispanic, and 18% identified as Black. Baseline mean (SD) SBP was 138.1 (11.2) mmHg, and baseline mean (SD) DBP was 84.3 (10.5) mmHg. In a modified intention-to-treat analysis, there was no significant difference in SBP (−4.8 mm Hg [95% CI: −10.8, 1.3, *p* = 0.123] and a −4.9 mmHg (95% CI: −8.6, −1.3, *p* = 0.009) greater mean DBP change in the intervention arm compared with the control arm at 12 weeks. Adverse events did not differ significantly between arms. The quadpill had a similar SBP and greater DBP lowering effect compared with candesartan 8 mg. Trial registration number: NCT03640312.

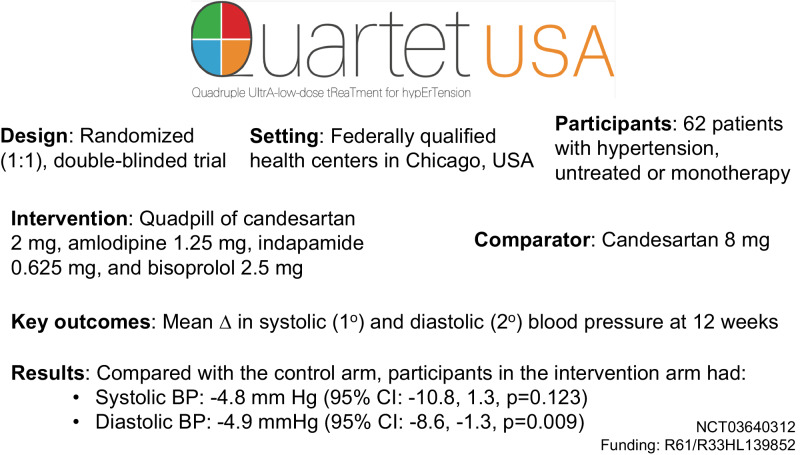

## Introduction

More than 1 billion adults have hypertension globally [1]. Despite widespread availability of generic blood pressure lowering drugs for decades, hypertension control rates (defined as a blood pressure <140/<90 mm Hg) remain persistently low (<50%) among adults in the United States [2]. Control rates are even lower (<25%) when accounting for newer, lower blood pressure targets (defined as a blood pressure <130/<80 mm Hg) recommended by national and international clinical practice guidelines [3–5]. Hypertension is more prevalent in racially and ethnically minoritized individuals, in whom control rates are also lower than other groups [6]. Most patients with hypertension are initially treated with a single blood pressure lowering drug that is titrated up over multiple, monthly office visits with additional medications added sequentially. Therapeutic inertia contributes to persistently low hypertension control rates and has not improved [7, 8]. Thus, a new approach is needed. New strategies are especially important among low-income individuals who seek care within federally qualified health centers, where the burden of hypertension is high and control rates are lower than the general population [9].

Previous trials of single drug ultra-low dose (i.e., one-quarter of a standard dose) blood pressure lowering therapy demonstrated an average of −4.7 systolic and −2.4 diastolic mm Hg greater blood pressure lowering compared with placebo with no significant difference in adverse events [10, 11]. Each additional drug added in a combination (e.g., two-, three-, and four-drug combinations) of quarter-dose blood pressure lowering drugs demonstrated a stepwise gradient of blood pressure lowering. Two- and three-drug single pill combinations have a favorable balance between greater blood pressure lowering effect, tolerability, adherence, and persistence in blood pressure control [12–14], As a result, major clinical practice guidelines and the World Health Organization recommend single pill combination therapy [3–5],

The QUARTET trial [15] in Australia randomized 591 adults with mild to moderate hypertension and demonstrated a mean −6.9/−5.8 mm Hg greater systolic/diastolic blood pressure lowering effect at 12 weeks with initiation of a four-drug, quarter-dose combination of irbesartan 37.5 mg, amlodipine 1.25 mg, indapamide 0.625 mg, and bisoprolol 2.5 mg (quadpill) compared with irbesartan 150 mg daily alone. This effect was observed even with add-on amlodipine 5 mg at six-week follow-up in either arm among individuals who were not controlled, defined as a blood pressure of 140/90 mm Hg or greater, while both arms remained blinded to initial treatment allocation. Most (>90%) participants in QUARTET identified as White or Asian. It is uncertain if similar effects would be observed in other race/ethnic groups based on previous reports of differential blood pressure lowering effects of some drug classes by race/ethnicity [16].

Thus, the objective of the QUARTET USA trial was to evaluate whether treatment with four-drug, quarter-dose combination therapy will have a greater reduction in office-measured blood pressure, and with fewer side effects, compared with standard dose monotherapy in patients with hypertension who receive care at a federally qualified health center network in Chicago, Illinois. We hypothesized that a quadpill would have a greater blood pressure lower effect than standard dose monotherapy without any increase in adverse events.

## Methods

### Ethics

The Northwestern Institutional Review Board provided oversight and approval for the trial. All study staff completed Good Clinical Practice training. Each participant provided informed consent prior to participation. The Food and Drug Administration approved the study drug for research purposes (Investigational New Drug: 133846), and an independent clinical trial monitor was employed to ensure compliance to Good Clinical Practice principles at the study sites. An independent Data and Safety Monitoring Board reviewed the study materials and provided interim guidance related to the safety, conduct, and analysis of the trial.

The methods for the QUARTET USA trial have been published [17]. Briefly, the study used a type I hybrid, phase II randomized (1:1), double-blind trial design to evaluate efficacy and safety of a quarter-dose combination of four blood pressure lowering drugs with a corresponding process evaluation to understand factors related to trial implementation and study drug acceptability. The results of the process evaluation have been reported separately [18].

### Study procedures

From August 2019 to May 2022, participants were recruited from two primary care health centers that are part of Access Community Health Network in Chicago, Illinois (ACCESS Ashland Family Health Center or ACCESS Martin T. Russo Family Health Center). Electronic health record data were used to screen potentially eligible participants followed by chart review by trained study staff. Clinicians also identified potentially eligible participants in person. Participants were recruited via telephone, electronic health record portal messaging, or in the health center. Study staff obtained informed consent among eligible individuals before starting study procedures. Data were captured using paper forms and transferred to REDCap, an electronic data capture system that was also used to perform randomization, stratified by clinic site. All investigators, study staff, and study clinicians were blinded except the study biostatistician (JDC) or her back-up team members (AC, MV, SDP).

After providing informed consent, participants reported demographic information and had their blood pressure measured in triplicate by trained study staff after a five-minute, unobserved rest period. Blood pressure was calculated using the mean of the second and third measurements. Staff used an appropriately sized cuff and a validated, automated blood pressure monitoring device (Omron HEM 907 Automated Blood Pressure Monitor). If participants remained eligible based on mean blood pressure measurements, then they completed baseline surveys, provided blood and urine samples, and had an electrocardiogram performed. Once eligibility was confirmed, then participants were randomized, and the study kit, including study drug, was dispensed. Participants who were on monotherapy at baseline were instructed to stop their treatment with no wash-out period.

At six-week follow-up, participants completed additional surveys, and study staff counted their pills, inquired about adverse events, and measured their blood pressure. If the systolic blood pressure was greater than 130 mm Hg or the diastolic blood pressure was greater than 80 mmHg, then participants in either arm were given open label amlodipine 5 mg daily in addition to continuing their blinded study medication.

Participants returned for the final study visit at 12 weeks and completed additional surveys and had their blood pressures measured. During the peak of the COVID-19 pandemic lockdown period, we provided active participants with an Omron Series 3 (*n* = 3) or Series 5 (*n* = 7) machine and trained them remotely on how to accurately measure their blood pressure in accordance with the principles of the study protocol (Supplementary Materials).

### Participants

Inclusion criteria were modified to simplify eligibility criteria during the COVID-19 pandemic and to align with the QUARTET trial in Australia more closely. Participants needed to be adults 18 years or older, English or Spanish speakers, and with mild to moderate hypertension that was either untreated or treated with monotherapy. Clinically measured blood pressure thresholds for inclusion were: 140–179/90–109 mm Hg for untreated participants and 130–159/85–109 mmHg for participants on monotherapy. Participants with contraindications to any of the included medications, prevalent cardiovascular disease, significant proteinuria, and secondary hypertension were not eligible. Women who were pregnant or were breastfeeding were also not eligible. Additional details related to eligibility are outlined in the Supplemental Materials.

### Intervention

We created a quarter-dose combination pill using a milling-and-filling approach that included candesartan 2 mg, amlodipine 1.25 mg, indapamide 0.625 mg, and bisoprolol 2.5 mg using Good Manufacturing Practice principles in collaboration with Sharp Clinical Services, which served as the study drug manufacturer. Candesartan was selected instead of irbesartan as in the original QUARTET trial to minimize pill size. Quarter doses are based on the usual maintenance doses outlined in the British National Formulary, Martindale, and Monthly Index of Medical Specialties [10]. Sharp also manufactured a matching active comparator pill (containing candesartan 8 mg only) and provided add-on amlodipine pills.

### Outcomes

The primary outcome was mean change from baseline in systolic blood pressure at 12 weeks, controlling for baseline. We report unadjusted and adjusted analyses that controlled for pre-specified covariates: sex, age, race/ethnicity, prior monotherapy, and limited literacy as defined by the Newest Vital Sign instrument [19]. Secondary outcomes included mean change in diastolic blood pressure, rates of hypertension control and medication adherence defined as 80% or greater use measured by pill count, and health related quality of life measured by the Patient-Reported Outcomes Measurement Information System (PROMIS) global physical and mental health outcomes.

Safety outcomes included occurrence of serious adverse events based on Good Clinical Practice definitions, relevant adverse drug effects (i.e., adverse events of special interest), and electrolyte abnormalities. Adverse events were collected by study staff during six- and 12-week follow-up visits and from participant contact during their time in the trial. Adverse event severity and relatedness were assessed by the blinded trial safety monitor. All events were coded by independent, blinded, and trained coders using the Medical Dictionary for Regulatory Activities (MedDRA) classification system [20]. Additional details related to the study outcomes are included in the Protocol and Statistical Analysis Plan (Supplementary Materials).

### Analysis

The primary study analysis used a linear mixed model with fixed study arm, study visit, and baseline outcome value effects and a random participant effect to account for within participant correlation. Secondary and safety analyses used generalized linear mixed modeling methods appropriate to the outcome of interest. For all outcomes, we used the model adjusted Wald type III tests for fixed effects to first evaluate significance of a visit-by-arm interaction at the 5% level of statistical significance. If insignificant at the 5% level, then this interaction term was removed and the model Wald type III test for fixed arm effect evaluated the overall intervention effect in this longitudinal model at the 5% level. Adverse event rates were tabulated overall and by arm, and chi-squared tests or exact methods were used to evaluate the differences across arms in event rates at the participant level.

No interim outcome analyses were planned. At the request of the Data and Safety Monitoring Board, we conducted a conditional power analysis in August 2021 using recruitment data from QUARTET USA trial in August 2021 and published trial data from QUARTET [15]. These analyses suggested that an analytic sample of 77 participants would provide 90% conditional power to detect a between-group difference of 5 mm Hg change in systolic blood pressure. We halted recruitment in May 2022 due to low recruitment.

Analyses involved the (modified) intention-to-treat (mITT) dataset, whereby all those participants with data at any follow-up time point and baseline were included in analyses according to arm to which they were randomized, regardless of adherence to the study protocol. We also conducted a sensitivity analysis on the per protocol dataset (defined as 80% treatment regimen adherence) since precise estimates of intervention effect (if any) on outcomes are important in a phase II study like this one. We used SAS (version 9.4, The SAS Institute) for all analyses. We defined statistical significance using a two‐sided *p* < 0.05 and did not include corrections for multiple hypothesis tests. MDH and JDC had full access to all data in the trial and take responsibility for its integrity and the data analysis.

## Results

**Fig. 1:**
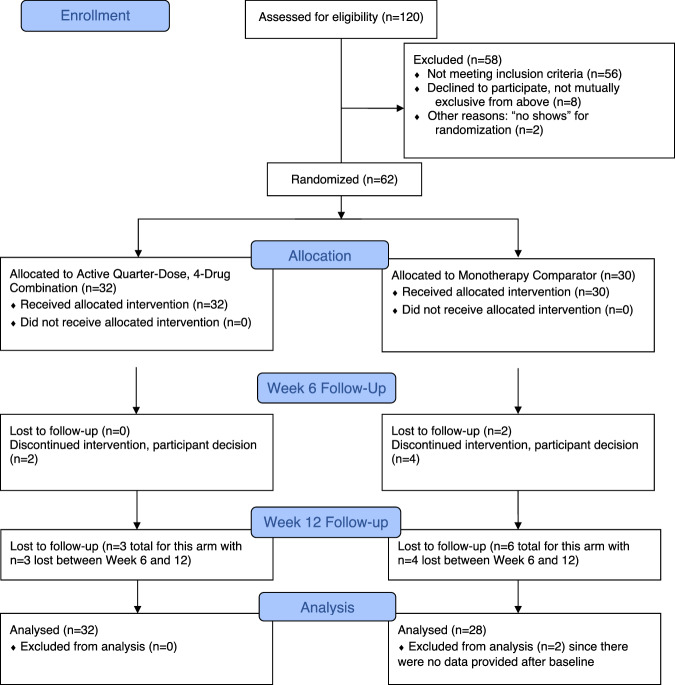
CONSORT flowchart of study participants

Figure 1 shows the flow of participants throughout the study, including reasons for exclusion. Among 120 participants assessed for eligibility, 62 were randomized, including 32 to the four-drug, quarter-dose combination therapy intervention arm and 30 to the control arm. Two participants in the control arm did not provide any follow-up data and were excluded from analysis.

Participants’ baseline characteristics are reported in Table 1 and were comparable between randomized arms. Mean (SD) age was 52 (11.5) years, 45% were female, 73% identified as Hispanic, and 18% identified as Black. Nearly half (48%) of participants did not have health insurance, 24% had an education level of less than grade nine, and 65% had an annual household income of $25,000 or less. Baseline mean (SD) systolic blood pressure was 138.1 (11.2) mm Hg, and baseline mean (SD) diastolic blood pressure was 84.3 (10.5) mm Hg. Most (84%) participants were on monotherapy for blood pressure lowering at baseline.

**Table 1:** Baseline characteristics of study participants

Characteristic, *n* (%)	Overall (*N* = 62)	Control (*N* = 30)	Intervention (*N* = 32)
Female sex	28 (45.2%)	13 (43.3%)	15 (46.9%)
Age, years, mean (SD)	52 (11.5)	52 (10.5)	52 (12.6)
Race/Ethnicity			
Hispanic	45 (72.6%)	21 (70.0%)	24 (75.0%)
Black	11 (17.7%)	7 (23.3%)	4 (12.5%)
White/Other	6 (9.7%)	2 (6.7%)	4 (12.5%)
No Health Insurance	30 (48.4%)	13 (43.3%)	17 (53.1%)
Education			
<Grade 9	15 (24.2%)	8 (26.7%)	7 (21.9%)
Grade 9–11	10 (16.1%)	4 (13.3%)	6 (18.8%)
High school/GED	22 (35.5%)	9 (30.0%)	13 (40.6%)
Undergraduate/AA degree	12 (19.4%)	7 (23.3%)	5 (15.6%)
Technical/vocational	3 (4.8%)	2 (6.7%)	1 (3.1%)
Employment status			
Full-time	25 (40.3%)	14 (46.7%)	11 (34.4%)
Part-time, retired, or homemaker	18 (29.0%)	10 (33.3%)	8 (25.0%)
Unemployed	19 (30.7%)	6 (20.0%)	13 (40.6%)
Household income between 0–$25,000 per year	40 (64.5%)	17 (56.7%)	23 (71.9%)
Married	30 (48.4%)	15 (50.0%)	15 (46.9%)
History of diabetes	17 (27.4%)	7 (23.3%)	10 (31.3%)
History of depression	16 (25.8%)	9 (30.0%)	7 (21.9%)
History of smoking	20 (32.3%)	9 (30.0%)	11 (34.4%)
Weekly alcohol use	19 (30.7%)	9 (30.0%)	10 (31.3%)
Body mass index, kg/m^2^, mean (SD)	34 (7.3)	34 (7.9)	33 (6.9)
Systolic blood pressure, mm Hg, mean (SD)	138 (11.2)	139 (10.8)	138 (11.8)
Diastolic blood pressure, mm Hg, mean (SD)	84 (10.5)	84 (11.5)	84 (9.6)
Heart rate, beats per minute, mean (SD)	72 (10.8)	72 (11.7)	72 (10.0)
Baseline blood pressure lowering monotherapy	52 (83.9%)	25 (83.3%)	27 (84.4%)

*AA* Associate in arts, *GED* General Educational Development, mm Hg Millimeters of mercury, *SD* Standard deviation

Table 2 shows the primary and selected secondary outcomes, including adjusted systolic and diastolic blood pressures and between-group differences in blood pressure at 12 weeks. At the end of the study period, the intervention arm had an adjusted mean blood pressure of 122 (95% CI: 118, 127)/73 (95% CI: 71, 76) mm Hg compared with the control arm adjusted mean blood pressure of 127 (95% CI: 123, 132)/78 (95% CI: 75, 81) mm Hg. Based on these differences, there was no significant difference in systolic blood pressure change (−4.8 mm Hg [95% CI: −10.8, 1.3, *p* = 0.123]) and a mean −4.9 mm Hg (95% CI: −8.6, −1.3, *p* = 0.009) greater diastolic blood pressure lowering in the intervention arm compared with the control arm. These findings were observed despite a substantially lower odds of amlodipine 5 mg add-on at six weeks in the intervention arm compared with the control arm (19% versus 53%, model estimated Odds Ratio = 0.08 [95% CI: 0.02, 0.41], *p* = 0.003). A higher proportion of participants achieved hypertension control defined as blood pressure <130/<80 mm Hg (66% versus 54%) in the intervention arm compared to the control arm, but the results were imprecise (model estimated Odds Ratio = 2.85 [95% CI: 0.94, 8.59], *p* = 0.063). Adherence to >80% of study medication was similar between arms (intervention: 66% versus control: 70%, model adjusted Odds Ratio = 0.63 [95% CI: 0.19, 2.08] *p* = 0.444).

**Table 2:** Primary and selected secondary outcomes: adjusted systolic and diastolic blood pressures and between-arm differences in blood pressure at 12 weeks, hypertension control at 12 weeks, add-on amlodipine at six weeks, and study drug adherence at 12 weeks

Outcome	Control model^a^ estimated mean (95% CI)	Intervention model^a^ estimated mean (95% CI)	Difference in least squared means^a^	*p*-value
Systolic blood pressure, mm Hg	127.1 (122.7, 131.5)	122.3 (118.2, 126.5)	−4.75 (−10.82, 1.32)	0.123
Diastolic blood pressure, mm Hg	77.9 (75.2, 80.6)	73.0 (70.5, 75.5)	−4.92 (−8.58, −1.27)	0.009

	Control, *N* (%)	Intervention, *N* (%)	Model-estimated odds ratios (95% CI)	*p*-value
Hypertension Control, week 12^b^	13 (54.2)	19 (65.5)	2.85 (0.94, 8.59)	0.063
Adverse event free + hypertension control, week 12^c^	8 (33.3)	5 (17.2)	0.83 (0.22, 3.16)	0.775
Amlodipine add-on, week 6	16 (53.3)	6 (18.8)	0.08 (0.02, 0.41)	0.003
Adherence^d^	21 (70.0)	20 (65.6)	0.63 (0.19, 2.08)	0.444

*CI* confidence intervals, *mm Hg* millimeters of mercury

^a^Models are adjusted for study arm, baseline and 6-week blood pressure, baseline age, sex, race/ethnicity (Hispanic), baseline monotherapy use, and baseline health literacy status (likely limited health literacy as measured by the Newest Vital Sign)

^b^Hypertension control defined as <130/ < 80 mm Hg

^c^Denominator for these percentages reflect just those with 12-week follow-up data: *N* = 24 for control arm and *N* = 29 for intervention arm. Hypertension control defined as <130/ < 80 mm Hg

^d^Adherence defined as >80% adherence to study medication as measured by pill counts

**Fig. 2:**
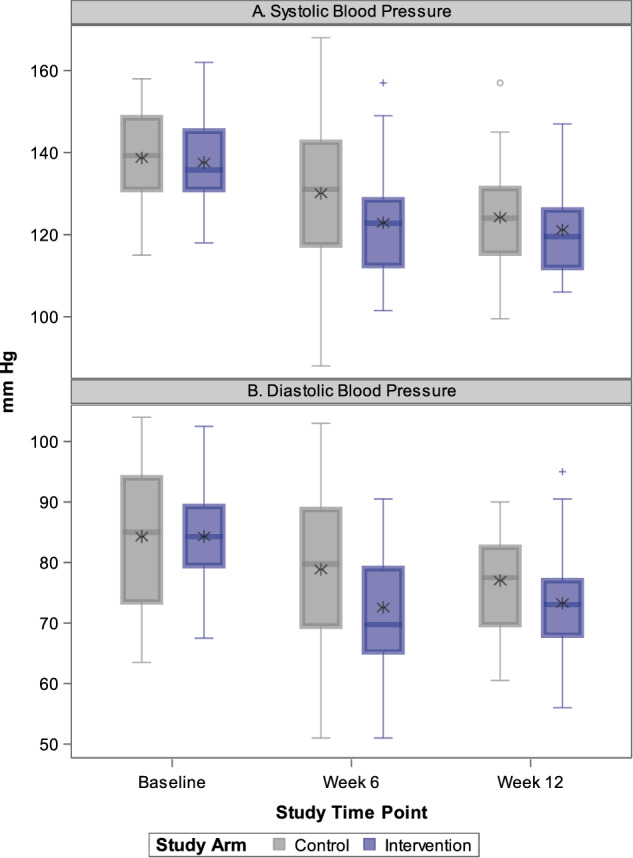
Box plots outlining temporal changes in distribution of systolic blood pressure (SBP) in (**A**) and distribution of diastolic blood pressures (DBP) by study arm in (**B**). Asterisk represents the mean value

Supplementary Table 1 shows the unadjusted blood pressure results, and Fig. 2 shows temporal changes in systolic and diastolic blood pressure between randomized arms from baseline to six- and 12-week follow-up. Supplementary Fig. 1a, b shows individual participant level changes in blood pressure. Mean blood pressures declined in both arms with greater declines from baseline to six-week follow-up in the intervention arm compared with the between-group difference observed during six- and 12-week follow-up.

Supplementary Fig. 2 shows results of pre-specified subgroup analysis, which do not provide evidence of heterogeneity of treatment effect by subgroups of baseline age, sex, race/ethnicity, baseline literacy level, and baseline monotherapy use.

Table 3 shows results related to adverse events among the study participants. Overall, there were 77 adverse events experienced by 34 participants. A somewhat higher proportion of participants experienced one or more adverse events in the intervention arm compared with the control arm (63% versus 47%, *p* = 0.210). Adverse events according to Medical Dictionary for Regulatory Activities (MedDRA) coding are shown in Supplementary Table 2. The proportion of patients who permanently discontinued the study drug due to adverse effects was low (intervention: *n* = 1 [3%] versus control: *n* = 3 [10%], exact *p* = 0.347). There were two serious adverse events, both experienced by participants randomized to the intervention arm, but neither was deemed related to the study drug by the blinded medical monitor. There were no significant between-arm differences in mean serum electrolyte, laboratory, and urine studies (Supplementary Table 3) nor health-related quality of life scores (Supplementary Table 4).

**Table 3:** Adverse events among study participants

Characteristic, No. (%)	Control (*N* = 30)	Intervention (*N* = 32)	*p*-value^a^
Any adverse event experienced	14 (46.7)	20 (62.5)	0.210
Any serious adverse event experienced	0 (100)	2 (6.3)	0.492^b^
Any events of special interest^c^	10 (33.3)	16 (50.0)	0.182
Any adverse event deemed at least possibly related to study drug	3 (10.0)	8 (25.0)	0.116
Discontinuation due to adverse event	3 (10.0)	1 (3.1)	0.347^b^

^a^*p*-values correspond to chi-squared likelihood ratio tests

^b^*p*-value corresponds to Fisher’s exact test result due to low cell counts

^c^Any events of special interest include any one of the side effect listed in the investigational brochure of each of the medications included in the quadpill: allergic reaction, anxiety, blurred vision/vision or hearing changes, breathing problems, change in sex drive/performance, chest pain, cold, tingling or numb hands/feet, confusion, cough, depression, diarrhea, dry mouth, dry or burning eyes, facial flushing, feeling faint/lightheaded/falling, headache, infection or flu-like symptoms, irregular/fast heartbeat, irregular/slow heartbeat, loss of appetite, muscle aches and pains, muscle cramps or spasms, nausea, vomiting, passing less urine, redness, blistering, peeling, loosening of skin (includes inside mouth), stomach gas, pain, sweating, swelling of hands or feet, swelling of legs or ankles, tremors, trouble sleeping, unusually weak or tired

## Discussion

The QUARTET USA results suggest that a strategy of initiating four-drug, quarter-dose combination therapy is similar in reducing systolic blood pressure and is more effective in lowering diastolic blood pressure than starting with standard dose angiotensin receptor blocker monotherapy at 12 weeks. These results were observed even with a substantially higher rate of add-on calcium channel blocker at six weeks in the control arm (53% versus 19%) among patients who receive care within a federally qualified health center network. The direction and magnitude of effect on systolic blood pressure were similar to the effect of diastolic blood pressure, but the observed effect was not statistically significant.

The four-drug, quarter-dose combination approach was developed to address poor blood pressure control rates, which have been exacerbated with lower treatment targets recommended by clinical practice guidelines [3, 4, 21], Contemporary guidelines increasingly recommend two-drug combination therapy, but this approach is often reserved for patients whose blood pressures are well above their treatment target [3]. QUARTET USA provides evidence that an ultra-low dose quadpill approach efficiently reduces blood pressure using a single pill as the initial treatment step. The mean blood pressure in the intervention arm at both six weeks (123/73 mm Hg) and 12 weeks (122/73 mm Hg) was below the target level recommended by contemporary clinical practice guidelines (blood pressure <130/ < 80 mm Hg). The upper bounds of the 95% confidence intervals for blood pressure in the intervention arm at 12 weeks (i.e., 126/75 mm Hg) in QUARTET USA suggest that this approach may be far more efficient in achieving lower targets than current approaches. For example, patients randomized in the intervention arm in the Systolic Blood Pressure Intervention Trial (SPRINT) needed three pills and six months to achieve a similar systolic blood pressure, a strategy that reduced the risk of major cardiovascular events by 25% and of all-cause mortality by 27% [22].

The results of the QUARTET USA trial are consistent with the QUARTET trial [15] results, which was conducted in Australia. We note some differences between the studies, including in mean baseline blood pressures (138/84 mm Hg in QUARTET USA versus 141/85 mm Hg in QUARTET), proportion of baseline monotherapy use (84% in QUARTET USA versus 46% in QUARTET), study drug adherence (67% at 12 weeks in QUARTET USA versus 76% at 52 weeks in QUARTET), and lower socioeconomic position among participants in QUARTET USA compared with QUARTET. QUARTET also used a higher blood pressure threshold for amlodipine 5 mg add on therapy at six weeks (140/90 mm Hg). Nevertheless, the similar direction and magnitude of effect from two trials with different study populations and drug combinations provides robust and supporting evidence for the overall approach of four-drug, quarter-dose combination therapy.

While there was a somewhat higher rate of adverse events in the intervention arm in the trial, the difference was not statistically significant. However, the rate of study drug discontinuation was lower in the intervention arm (3% vs. 10%). There were only two serious adverse events, and while both occurred among participants in the intervention arm, neither was deemed related to the study drug. Reassuringly, safety measures of electrolyte and serum creatinine levels were not significantly different between groups. The overall rate of discontinuation due to adverse events at 12 weeks was higher in QUARTET USA compared with QUARTET (7% versus 3%). Differences in study populations, sample size, and methods of adverse event ascertainment may have influenced these results. On the other hand, the rates of serious adverse events were low and similar in both trials (3% versus 2%). In aggregate, this suggests a clinically meaningful and favorable risk-benefit balance of a four-drug, quarter-dose combination therapy approach to safely, effectively, and efficiently lower blood pressure to achieve treatment goals.

QUARTET USA enrolled a large proportion of participants who self-identified as Hispanic or Black with low education levels and household income from a federally qualified health center network in Chicago, Illinois. More than 30 million patients receive care in federally qualified health centers in the U.S., which receive federal funding under Section 330 of the Public Health Service Act to care for vulnerable populations [23]. While patients from minoritized populations are disproportionately affected by hypertension [9], they are less likely to be included in clinical trials to address this condition [24]. The design of the QUARTET USA trial is thus responsive to the burden of hypertension in the United States, and the results inform treatment strategies to reduce racial and ethnic and socioeconomic disparities in hypertension control [20, 25].

To support wider implementation of a four-drug, quarter-dose combination therapy approach, our study team also conducted an explanatory sequential, mixed methods process evaluation of the trial among patients and healthcare professionals [17]. This treatment approach was considered acceptable and convenient, despite the tension that patients reported related to necessity and concerns of blood pressure lowering medications. Healthcare professionals expressed some concerns about relative inflexibility of the treatment regimen, which may paradoxically lead to greater therapeutic inertia among patients treated with fixed dose combination therapy [26]. More importantly, process evaluation participants said that health insurance coverage and limiting out-of-pocket costs for four-drug, quarter-dose combination were essential for future sustainment and scale-up. Our results are generally similar to those from previous process evaluations of combination therapy for prevention and control of cardiovascular diseases [27, 28].

QUARTET USA had several strengths, including using a randomized, double-blind trial design and an active, well-tolerated comparator. Blood pressure was measured using rigorous and reliable methods to maximize precision, and the study included participants from diverse backgrounds in a resource-limited context. The trial was also conducted during the COVID-19 pandemic, which had a major impact on clinical and research activities. However, adherence to study procedures was high, and loss to follow-up was relatively low.

### Limitations

Despite these strengths, the trial had several limitations, including lower than planned recruitment and study drug adherence, which likely reduced the statistical power to detect an effect on the primary outcome of between-arm difference in systolic blood pressure. There were changes to the study protocol to respond to the COVID-19 pandemic, and some planned outcome measures, such as 24-h ambulatory blood pressure became infeasible to collect. Details of these changes are outlined in the study protocol. Nevertheless, the results add to the body of literature of quarter-dose, four drug combination therapy [29, 30], and our team will participate in a pooling of published trials to better characterize effect sizes, including in different contexts, using different combinations, and across subgroups.

## Conclusions

The QUARTET USA trial provides evidence of efficacy and safety of a four-drug, quarter-dose combination therapy with a similar reduction in systolic blood pressure and a greater reduction in diastolic blood pressure compared with standard dose angiotensin receptor blocker monotherapy in patients with mild to moderate hypertension receiving care at a federally qualified health center network in Chicago, Illinois. This new approach may be useful in improving blood pressure control among the more than one billion adults with hypertension around the world, especially among vulnerable groups in whom prior research has been limited.

### Supplementary information

Supplemental Materials

Protocol

Statistical Analysis Plan
