# Supplementary material for: Efficacy and safety of a four-drug, quarter-dose treatment for hypertension: the QUARTET USA randomized trial
Source: Hypertens Res. 2024 Apr 8;47(6):1668–77. doi: 10.1038/s41440-024-01658-y (PMC11150153; doi:10.1038/s41440-024-01658-y)

Supplemental Materials

1. Supplemental Tables and Figures
2. Protocol
3. Statistical Analysis Plan

Supplementary Table 1. Unadjusted systolic and diastolic blood pressure, heart, and proportion of hypertension control at baseline and six- and 12-week follow-up by study arm.

Supplementary Table 2. Adverse events among study participants according to Medical Dictionary for Regulatory Activities (MedDRA) coding.

Supplementary Table 3. Laboratory data, overall and by study arm.

Supplementary Table 4. Health related quality of life, overall and by study arm.

Supplementary Figure 1. Spaghetti plots of individual-level changes in systolic blood pressure (A) and diastolic blood pressure (B) from baseline to six- and 12-week follow-up by study arm.

Supplementary Figure 2. Subgroup analyses to evaluate potential heterogeneity of treatment effect by subgroup baseline age, sex, ethnicity, health literacy level, and baseline monotherapy use. The solid vertical line provides a reference at ‘0’ (no effect), and the dotted vertical line illustrates the model-estimated mean main effect across arms.

Supplementary Table 1. Unadjusted systolic and diastolic blood pressure, heart rate, and proportion of (adverse event free) hypertension control at baseline and 6- and 12-week follow-up by study arm.

		Baseline		Week 6		Week 12	
**Measure, Mean (SD)**	**Arm**	**N**	**Result**	**N**	**Result**	**N**	**Result**
Systolic Blood Pressure, mm Hg	Control	30	138.68 (10.75)	28	130.09 (18.78)	24	124.21 (12.60)
	Intervention	32	137.59 (11.78)	32	122.88 (13.14)	29	121.17 (11.86)
Diastolic Blood Pressure, mm Hg	Control	30	84.28 (11.54)	28	78.86 (14.23)	24	77.02 (7.72)
	Intervention	32	84.31 (9.57)	32	72.56 (9.77)	29	73.29 (8.95)
Change Systolic Blood Pressure, mm Hg	Control		NA	28	-8.89 (17.03)	24	-14.42 (13.94)
	Intervention		NA	32	-14.72 (16.23)	29	-15.90 (12.18)
Change Diastolic Blood Pressure, mm Hg	Control		NA	28	-5.68 (9.33)	24	-8.23 (10.44)
	Intervention		NA	32	-11.75 (11.81)	29	-11.21 (7.74)
Heart Rate, beats per minute	Control	30	71.73 (11.65)	28	73.02 (13.16)	24	71.56 (13.43)
	Intervention	32	71.52 (10.03)	32	67.91 (11.00)	29	68.00 (11.61)
Blood Pressure Controlled, No. (%)	Control	30	3 (10.00)	28	11 (39.29)	24	13 (54.17)
	Intervention	32	2 (6.25)	32	22 (68.75)	29	19 (65.52)
Adverse Event Free + Blood Pressure Controlled, No. (%)	Control		NA	28	5 (17.86)	24	8 (33.33)
	Intervention		NA	32	8 (25.00)	29	5 (17.24)

mm Hg: millimeters of mercury, NA=not applicable

Supplementary Table 2. Adverse events among study participants according to Medical Dictionary for Regulatory Activities (MedDRA) coding.

Adverse Events by MedDRA System Organ Class	Arm					
	Control		Intervention		Overall	
	N	%	N	%	N	%
**Overall**	30	100	47	100	77	100
Cardiac disorders	1	3.33	0	0	1	1.30
Ear and labyrinth disorders	0	0	1	2.13	1	1.30
Eye disorders	1	3.33	2	4.26	3	3.90
Gastrointestinal disorders	6	20.00	8	17.02	14	18.18
General disorders and administration site conditions	6	20.00	8	17.02	14	18.18
Injury, poisoning and procedural complications	0	0	1	2.13	1	1.30
Investigations	3	10.00	3	6.38	6	7.79
Musculoskeletal and connective tissue disorders	2	6.67	1	2.13	3	3.90
Nervous system disorders	2	6.67	10	21.28	12	15.58
Psychiatric disorders	1	3.33	2	4.26	3	3.90
Respiratory, thoracic and mediastinal disorders	6	20.00	5	10.64	11	14.29
Skin and subcutaneous tissue disorders	1	3.33	2	4.26	3	3.90
Vascular disorders	1	3.33	4	8.51	5	6.49

Supplementary Table 3. Laboratory data, overall and by study arm.

	Baseline		Week 6		Week 12	
**Lab Marker, mean (SD)**	**Control**	**Intervention**	**Control**	**Intervention**	**Control**	**Intervention**
Serum sodium, mmol/L	140.9 (2.0)	140.5 (2.5)	140.7 (1.8)	139.7 (2.5)	140.4 (1.7)	139.9 (2.2)
Serum potassium, mmol/L	4.4 (0.4)	4.4 (0.3)	4.6 (0.3)	4.3 (0.3)	4.5 (0.4)	4.4 (0.4)
Serum glucose, mg/dL	111.6 (42.0)	127.6 (77.7)	115.7 (48.4)	129.4 (79.2)	118.4 (45.8)	109.5 (40.5)
Serum creatinine, mg/dL	0.8 (0.2)	0.8 (0.2)	0.8 (0.3)	0.8 (0.2)	0.8 (0.2)	0.8 (0.2)
Urine albumin to creatinine ratio, mg/g	23.5 (34.8)	24.5 (39.3)	12.2 (10.1)	20.4 (37.2)	13.5 (17.1)	12.2 (13.2)
Serum uric acid, mg/dL	4.8 (1.3)	5.2 (1.6)	5.0 (1.3)	5.7 (1.7)	4.5 (1.1)	5.7 (1.5)
Serum total cholesterol, mg/dL	182.0 (42.9)	198.2 (39.9)	171.5 (30.4)	192.9 (38.1)	168.5 (34.4)	197.3 (40.6)

SD: standard deviation

Supplementary Table 4. Health related quality of life at baseline and 12-week follow-up, by study arm.

PROMIS Domain, Mean (SD)	Baseline Control N=25	Baseline Intervention N=29	12-week follow-up Control N=25	12-week follow-up Intervention N=29
Physical Health T-Score	41.4 (8.1)	46.4 (7.9)	47.4 (9.8)	47.7 (8.6)
Mental Health T-Score	45.2 (8.9)	47.1 (7.9)	48.0 (10.4)	50.2 (8.5)

T-score=50 represents the 50th percentile compared with a normative sample. Table includes only those with data at follow-up for this outcome.

Supplementary Figure 1. Spaghetti plots of individual-level changes in systolic blood pressure (A) and diastolic blood pressure (B) from baseline to six- and 12-week follow-up by study arm.

A. B.

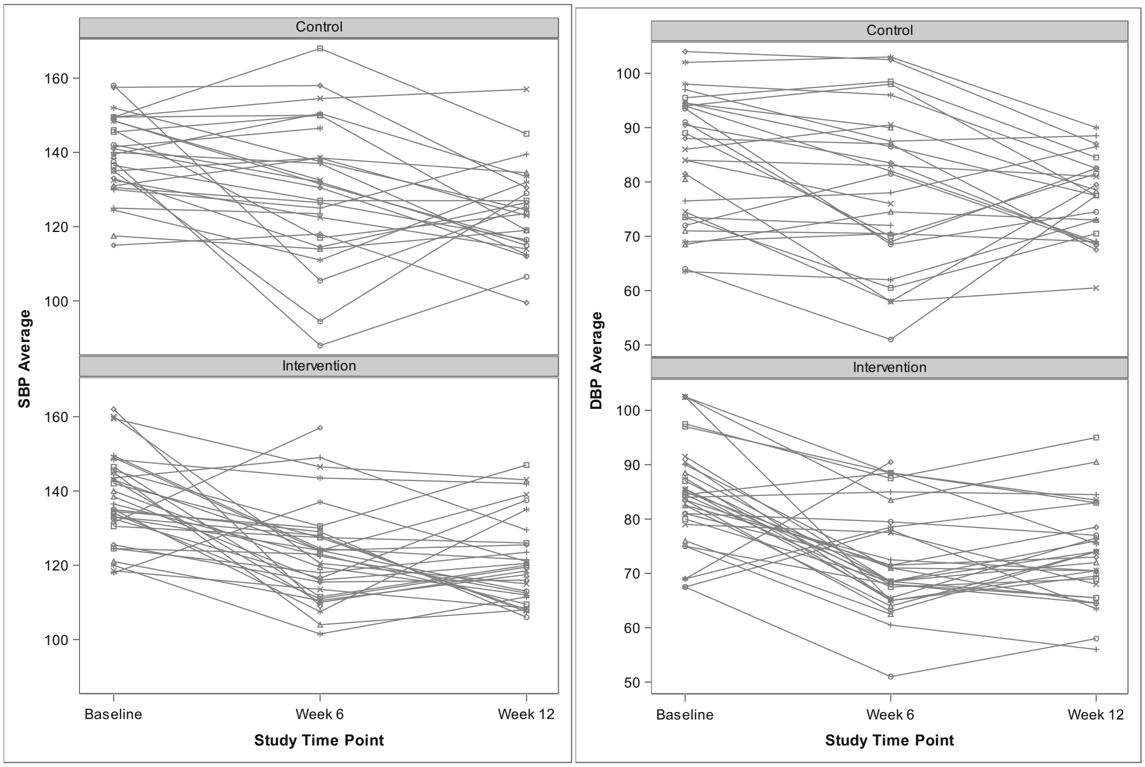

SBP: systolic blood pressure; DBP: diastolic blood pressure

Supplementary Figure 2. Subgroup analyses to evaluate potential heterogeneity of treatment effect by subgroup baseline age, sex, ethnicity, literacy level, and baseline monotherapy use.

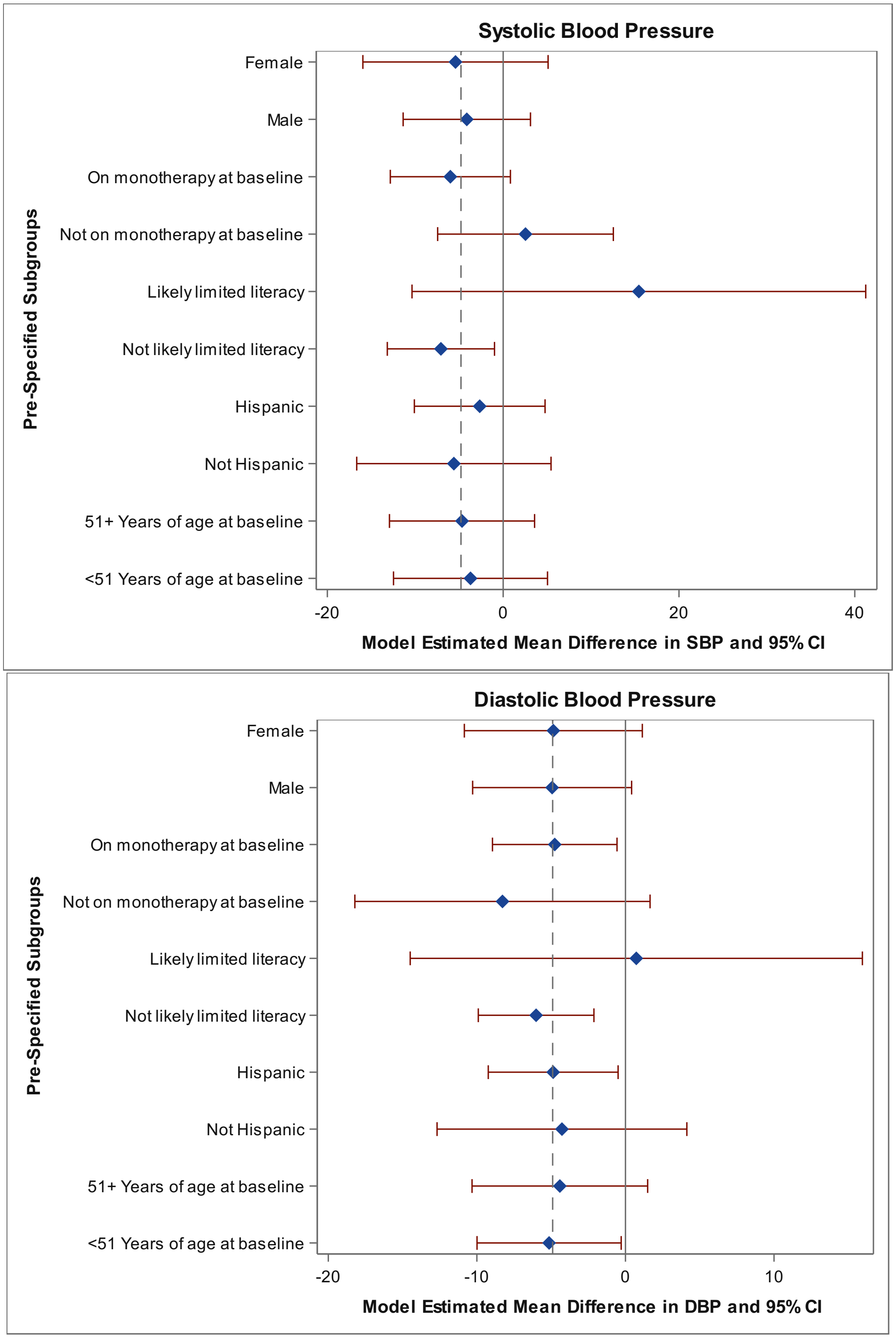

Supplement: Supplementary file 1 — Supplemental Materials [file 41440_2024_1658_MOESM1_ESM.pdf]
