# Supplementary material for: Efficacy and safety of a four-drug, quarter-dose treatment for hypertension: the QUARTET USA randomized trial
Source: Hypertens Res. 2024 Apr 8;47(6):1668–77. doi: 10.1038/s41440-024-01658-y (PMC11150153; doi:10.1038/s41440-024-01658-y)
Supplement: Supplementary file 2 — Protocol [file 41440_2024_1658_MOESM2_ESM.pdf]

PROTOCOL TITLE: A double blind randomized controlled trial to assess the efficacy and safety of a quadruple ultra-low-dose treatment for hypertension (QUARTET USA).

CO-PRINCIPAL INVESTIGATORS:

Mark Huffman, MD, MPH (contact PI)

Professor of Medicine

Division of Cardiology

Washington University School of Medicine

660 S. Euclid Ave., MSC 8086-43-13

St. Louis, MO 63110

(314) 747-9487

Email: m.huffman@wustl.edu

Jody Ciolino, PhD

Assistant Professor in Preventive Medicine and Medicine

Northwestern University Feinberg School of Medicine

680 N. Lake Shore Drive, Suite 1400

Chicago, IL 60611

Tel: +1-312-503-3980

Fax: +1-312-908-9588

Email: jody.ciolino@northwestern.edu

VERSION NUMBER: 6.1

VERSION DATE: April 20, 2022

STUDY SUMMARY:

Investigational Agent(s)<br>(Drugs or Devices)	Active Treatment: Low Dose Quadruple Combination Therapy (LDQT) comprising candesartan 2 mg, amlodipine 1.25 mg, indapamide 0.625 mg, and bisoprolol 2.5 mg<br>Comparator: Candesartan 8 mg
IND / IDE / HDE #	133846
Indicate<br>Special Population(s)	☐ Children<br>☐ Children who are wards of the state<br>☐ Adults Unable to Consent<br>☐ Cognitively Impaired Adults<br>☐ Neonates of Uncertain Viability<br>☐ Pregnant Women<br>☐ Prisoners (or other detained/paroled individuals)<br>☐ Students/Employees
Sample Size	87
Funding Source	National Heart, Lung, and Blood Institute
Indicate the type of consent to be obtained	☒ Written<br>☐ Verbal/Waiver of Documentation of Informed Consent<br>☐ Waiver of HIPAA Authorization<br>☐ Waiver/Alteration of Consent Process
Site	☒ Lead Site (For A Multiple Site Research Study)<br>☐ Data Coordinating Center (DCC)
Research Related Radiation Exposure	☐ Yes<br>☒ No
DSMB / DMC / IDMC	☒ Yes<br>☐ No

ABBREVIATIONS:

ABPM	Ambulatory Blood Pressure Monitoring
ACE-I	Angiotensin-converting-enzyme inhibitor
AE	Adverse Event
ALP	Alkaline Phosphatase, a kind of enzyme
ALT	Alanine Transaminase, a kind of enzyme
ARB	Angiotensin II receptor blocker
AST	Aspartate Aminotransferase, a kind of enzyme
BB	Beta Blocker
BMI	Body Mass Index
BP	Blood Pressure
CABG	Coronary Artery Bypass, a type of surgery that improves blood flow to the heart
CAD	Coronary Artery Disease
CCB	Calcium Channel Blocker
CRF	Case Report Form
DBP	Diastolic Blood Pressure
DSMB	Data and Safety Monitoring Board
DSMP	Data and Safety Monitoring Plan
ECG/EKG	Electrocardiogram
eConsent	Electronic consent
ECT	Electroconvulsive Therapy, a medical treatment used in patients with severe major depression
eGFR	Estimated Glomerular Filtration rate, a number based on the creatinine level in the blood
GCP	Good Clinical Practice
GGT	Gamma-Glutamyl Transferase, a kind of enzyme found in many organs throughout the body
HDL	High Density Lipoprotein, a group of lipoproteins that transport all fat molecules around the body in the extracellular water
IPD	Individual Participant Data
LVH	Left Ventricular Hypertrophy, a term for a heart’s left pumping chamber that has thickened and may not be pumping efficiently
MI	Myocardial Infarction, a kind of heart attack
PCI	Percutaneous Coronary Intervention, a non-surgical procedure to treat narrowing of the coronary arteries
PROMIS	Patient-reported Outcome Measurement Information System
QRS	The main spike seen on an ECG line
QTc	The interval that represents the total time from ventricular depolarization to complete repolarization
SAE	Serious Adverse Event
SBP	Systolic Blood Pressure
TIA	Transit Ischemic Attack, a brief stroke-like attack that can be resolved within minutes to hours
UACR	Urine Albumin: Creatinine Ratio

1. OBJECTIVES

To investigate, in a double-blind randomized controlled trial, whether initiating treatment with ultra-low-dose quadruple-combination therapy ("LDQT") will lower office blood pressure more effectively, and with fewer side effects, compared to initiating standard dose monotherapy in patients with hypertension.

Primary hypothesis: A combination pill comprising four types of blood pressure lowering medications, each at one-quarter standard doses, will lower office blood pressure more effectively than initiating patients with standard dose monotherapy in patients with hypertension.

2. BACKGROUND

2.1. Burden of raised blood pressure and treatment gaps

Elevated blood pressure, also known as hypertension when $\geq$130/$\geq$80 mmHg, is a leading cause of preventable morbidity and mortality globally.[1] In the United States, approximately 45.6% of adults, or 103.3 million individuals have hypertension, and prevalence rates are rising, particularly among non-Hispanic black men and women in whom prevalence rates exceed 50%.[2] Among Americans with elevated blood pressure, 81.9 million are eligible for pharmacotherapy based on the 2017 AHA/ACC blood pressure guideline, an absolute increase of 4.2 million Americans compared with previous thresholds for initiation of pharmacotherapy ($\geq$140/$\geq$90 mmHg). The benefits of blood pressure lowering in reducing cardiovascular events are unequivocal. Blood pressure lowering therapy leads to an estimated 11% reduction in all-cause mortality (relative risk [RR] = 0.89 [95% CI 0.85, 0.95]), 16% reduction in coronary heart disease events (RR = 0.84 [95% CI 0.79, 0.90]), and 36% reduction in stroke (RR = 0.64 [95% CI 0.56, 0.73]). The typical estimated blood pressure lowering effect from a standard dose of a single drug ranges from 6-9/3-6mmHg.[3] Therefore, improving blood pressure control is a strategic priority for reducing the burden of cardiovascular disease in the United States[2] and around the world.[1]

While most Americans are aware of their hypertension and while most of those who are aware receive treatment (75%), less than half (47%) are controlled using a goal blood pressure of <130/<80 mmHg when using nationally representative data (compared with 52% based on the previous target of <140/<90 mmHg).[2] These recommendations were based on high-quality data from the Systolic Blood Pressure Intervention Trial (SPRINT),[3,4] yet the method of implementing this strategy, which includes repeated clinic visits with repeated dose titrations of individuals drugs, is challenging. This strategy is also limited because maximizing doses of individual drugs leads to a greater risk of side effects without a similar benefit to blood pressure reduction.

This proposal aims to substantially simplify and improve the efficacy and safety of blood pressure-lowering treatment by changing the initial treatment paradigm for patients with essential hypertension who are eligible for pharmacotherapy without prevalent cardiovascular disease. We hope to change the treatment paradigm from moderate dose, single-drug therapy with repeated dose titration to ultra-low dose, quadruple combination therapy (LDQT). Combination therapy improves adherence in many chronic conditions, including elevated blood pressure[5] and cardiovascular diseases,[6] but it has been typically reserved for individuals with very high (systolic blood pressure [SBP] >150 mmHg in the 2017 guideline compared with >160

mmHg in previous guidelines) or difficult to control blood pressures.[3] The proposed treatment regimen has the potential to make it easier for typical patients and clinicians to achieve blood pressure goals with fewer pills and fewer clinic visits to prevent fatal and non-fatal cardiovascular disease events and to improve cardiovascular health with a focus on vulnerable groups who bear a disproportionate burden of hypertension and hypertension-related diseases.

2.2. Barriers to hypertension control

There are multiple barriers to hypertension control, including patient-and physician-related factors. Patient adherence is a major factor in hypertension control and is worsened by increased number of medications, complexity of dosing regimens, and medication side effects.[8] "Therapeutic inertia", which is the reluctance of clinicians to treat "mild" hypertension (SBP <160 mmHg) despite demonstrated benefits[7] or to up-titrate medications, remains an important barrier to hypertension control. Current guidelines typically recommend initiating monotherapy, up-titration, switching drugs if not tolerated, and adding other agents if needed.[3] This strategy often takes multiple visits to achieve blood pressure goals, which results in most patients remaining on monotherapy with inadequate blood pressure control. SPRINT participants randomized to a SBP goal <120 mmHg took, on average, three pills at standard dose for blood pressure control compared with two pills at standard dose among individuals randomized to the SBP goal <140 mmHg.[4] Translating these important research findings into routine clinical practice, particularly among vulnerable populations, will be difficult using the current paradigm of blood pressure management.

2.3. Combination therapy for blood pressure lowering

Because 50% to 75% of patients require multi-drug treatment for blood pressure control, there has been increasing interest in the initial use of combination therapy. A 2013 survey of 31 international hypertension guidelines demonstrated that 27 (87%) of guidelines recommend use of combination for initial treatment but typically only as an option for patients at >20/10 mmHg from goal,[8] which is concordant with the 2017 AHA/ACC blood pressure guideline. Nevertheless, there is evidence of improved adherence for combination-based therapy (albeit typically two drug combinations) compared to monotherapy for treatment of elevated blood pressure, cardiovascular diseases, and other chronic conditions with estimates ranging from 25% to 50%.[6] Most recently, the TRIple pill vs. Usual care Management for Patients with mild-to-moderate Hypertension (TRIUMPH) trial evaluating half-dose, triple drug combination (telmisartan 20 mg, amlodipine 2.5 mg, chlorthalidone 12.5 mg) demonstrated hypertension control rates of 70% in the intervention compared with 55% in the usual care arm at six months among 700 adults in Sri Lanka.[9]

While these data are promising for the use of low-dose combination therapy for the treatment of mild and moderate hypertension, they need further study with an active comparator arm to reduce the risk of performance bias and within a broader, more diverse population. Combinations with more drugs but at lower doses (four drugs at quarter doses) than what was used in TRIUMPH (three drugs at half doses) may be even more effective and with a better side effect profile. Therefore, there is a clear need for improved strategies to: 1) make treatment of hypertension more effective and easier to implement for patients and clinicians, 2) quickly and

safely bring elevated blood pressure to lower goals than those recommended in the past, and 3) increase long-term adherence with therapy.

In August 2021, the QUARTET (Australia) trial compared the effect of irbesartan at 37.5 mg, amlodipine at 1.25 mg, indapamide at 0.625 mg, and bisoprolol at 2.5 mg with irbesartan 150 mg daily among 591 patients with mild to moderate hypertension who had a baseline mean (SD) systolic blood pressure of 142 (13) mmHg in the intervention group and 140 (13) mmHg in the control group.[10] Compared with the irbesartan control group, patients randomized to the quadpill had a 6.9 mmHg (95% CI: 4.9-8.9) greater systolic blood pressure lowering at 12 weeks. These results were sustained up to 12 months in a sample of 417 patients who were followed for 12 months when the difference was 7.7 mmHg (95% CI: 5.2-10.3) of systolic blood pressure. Dizziness was reported among 93 (31.0%) patients in the intervention group compared with 74 (25.4%) patients in the control group (RR 1.27 [95% CI: 0.98-1.64]). There were no serious adverse events due to syncope, falls, or acute kidney injury. The study participants were largely White (82%) and Asian (12%) race/ethnicity. Thus, there is promising evidence of an ultra-low-dose combination approach, but additional data are needed in more diverse populations.

3. STUDY ENDPOINTS

3.1. Primary

Mean change (from baseline) in automated office SBP at 12 weeks adjusted for baseline values.

3.2. Secondary

1. Mean automated office SBP at 12 weeks adjusted for baseline values.
2. Other blood pressure measures in active vs. control groups (adjusted for baseline values in each case):
    a. Mean change (from baseline) in automated office SBP at six weeks.
    b. Mean automated office SBP at six weeks.
    c. Mean change (from baseline) in automated office DBP at six and 12 weeks.
    d. Mean automated office DBP at six and12 weeks.
    e. Proportion of patients with hypertension control (percent with SBP < 130 mmHg and DBP <80 mmHg) at six and 12 weeks.
    f. Proportion of patients requiring step-up treatment (ever, and at each study time point).
    g. Proportion of patients with adverse event-free hypertension control (percent with SBP < 130 mmHg and DBP < 80 mmHg).

3. Medical adherence:
    a. Medication adherence defined by objective pill counts.

4. Health-related quality of life:
    a. Mean change (from baseline) in health-related quality of life using PROMIS Global Health instrument.

3.3. Tolerability & Safety

a. Percentage of participants with any SAE according to GCP definition.
b. Percentage of participants with any potentially relevant side effect (refer to the adverse event case report form and the list of relevant side effects from the informed consent form, included as an Appendix).
c. Rate of relevant side effects at the participant level.
d. Mean change (from baseline) in continuous serum potassium (mEq/L).
e. Mean change (from baseline) in continuous serum sodium (mEq/L).
f. Mean change (from baseline) in continuous blood urea nitrogen (mg/dl).
g. Mean change (from baseline) in continuous serum creatinine (mg/dl).

3.4. Exploratory

1. 24-hour ambulatory blood pressure measures
    a. Mean 24-hour SBP and DBP assessed through 24-hour ambulatory blood pressure monitoring.
    b. Mean daytime (0600 to 2200) SBP and DBP assessed through 24-hour ambulatory blood pressure.
    c. Mean nighttime (2200 to 0600) SBP and DBP assessed through 24-hour ambulatory blood pressure.
    d. Proportion of dippers* assessed through 24-hour ambulatory blood pressure.
    e. Mean daytime SBP and DBP load assessed through 24-hour ambulatory blood pressure. Load is defined as the percentage of abnormally elevated readings; daytime SBP / DBP elevated readings would be 130 / 80 mmHg or above.
    f. Mean nighttime SBP and DBP load assessed through 24-hour ambulatory blood pressure. Abnormally elevated readings for nighttime SBP / DBP would be 120 / 70 mmHg or above.
    g. Percentage of participants with morning surge, calculated as the difference between the mean SBP during the morning hours and nighttime trough SBP. Trough SBP was defined as the mean of three SBP measurements: the lowest nighttime SBP and the measurements immediately preceding and following this measurement.[11]
    h. Coefficient of variation of SBP and DBP assessed through 24-hour ambulatory blood pressure as defined as the ratio of the 24-hour standard deviation of BP / mean 24-hour value.
    i. Day-night variability (SDdn), which uses the SD for daytime measurements and, separately for nighttime measurements, to calculate a weighted mean of these SDs.[12]
    j. Average real variability (ARV), which is calculated as the average absolute difference between consecutive readings over the 24-hour ABPM period.[12]
    k. Additional exploratory outcomes will be assessed as needed to inform individual participant data (IPD) meta-analysis with international QUARTET studies.

*Dipping is defined as nighttime blood pressure falling more than 10% from the daytime values OR night / day blood pressure ratio less than 0.9 and greater than 0.8 with normal diurnal blood pressure pattern.[11]

4. STUDY INTERVENTION

Patients who are eligible to participate in the trial will be randomized (1:1 allocation) to one of two study arms, and they will take daily:

1. Low Dose Quadruple Therapy (LDQT) comprising candesartan 2 mg, amlodipine 1.25 mg, indapamide 0.625 mg, and bisoprolol 2.5 mg
   OR
2. Candesartan 8 mg

The LDQT includes quarter standard doses of four blood pressure lowering medications, which are currently approved by the US Food and Drug Administrations for the treatment of hypertension. The active comparator follows current clinical practice guidelines for the majority of Americans, initiating treatment with an angiotensin II receptor blockers (ARB), which has the best side effect profile of any blood pressure lowering therapy. In both arms, if blood pressure is not controlled (SBP > 130 mmHg or DBP > 80 mmHg) at the six-week follow-up assessment, then study staff will provide participants with an open label pill of amlodipine 5 mg to supplement their study drug, which may be relevant to participants from some race/ethnic groups, including African-Americans. The combination of a calcium channel blocker with an ACE-inhibitor has been demonstrated to be more effective in reducing cardiovascular events than the combination of a thiazide-like diuretic with an ACE-inhibitor based on the ACCOMPLISH trial.[13]

All study drugs, including LDQT, active comparator, and amlodipine add-on will be stored, controlled, and administered centrally. Control of drugs is described in full in the detailed Manual of Procedures. Drug product used in this study is subject to regulatory review and approval through an investigator-initiated investigational new drug (IND) process. Prior to use in human subjects, IND approval will be required.

5. PROCEDURES INVOLVED

QUARTET USA is a phase II, two-arm, randomized (1:1 allocation), double-blind clinical trial. Study participation involves the following study visits: (1) a two-day screening and baseline visit (including 24-hour ABPM and randomization if eligible), (2) six-week follow-up visit, and (3) 12-week follow-up visit. We do not plan for any long-term follow-up data at this time. After participants complete all procedures in their 12-week visit and return their ABPM, they will exit the study, and we plan to stop data collection for them at that time.

To monitor safety, we will document all adverse events in case report forms (CRFs) with expedited reporting for adverse events meeting criteria as defined by regulatory authorities (e.g., MedWatch form for SAEs that are probably or definitely related to the study drug and unexpected). We plan to perform centralized monitoring of all essential study data with targeted on-site visits as appropriate (refer to the DSMP and Adverse Event Reporting Plan for details). An external DSMB will also review all essential outcome and safety data every six months at minimum.

The following study visit matrix explains the research procedures and assessments / data collected at each visit. Full description of procedures and assessments is included within the Manual of Procedures.

**REDCap Event**			
**Study Timeline**	**Screening and Randomization**	**6-Week Follow-up Visit**	**12-Week Follow-up Visit**
Visit Window from Previous Visit	-12 weeks	+/- 7 days	+/- 7 days
Location	Clinic with telehealth screening and eConsent option	Clinic	Clinic
**Study Activities**			
Informed consent	X[1]		
Demographics[2]	X[1]		
Automated office (clinic) blood pressure measured in the previous 12 weeks	X[1]		
Blood pressure and heart rate measurements, by research team	X	X	X
ECG	X[3]		
Anthropometrics and Medical history	X[1]		
Concomitant medications	X[1]	X	X
Lifestyle questions	X[1]		
PROMIS global health	X[1]		X
Laboratory assessments[4]	X[3]	X	X
24-hour ABPM	X		X
Inclusion and exclusion criteria	X[1]		
Randomization	X		
Study Drug dispense	X	X	
Study Drug return		X	X
Medication adherence		X	X
Participant status		X	X
Health service utilization		X	X
AEs and SAEs	X	X	X

[1]This form may be completed electronically via a telehealth visit up to 90 days before the date of randomization (12 weeks in the case of previous automated office blood pressure measurement).

[2]Such as date of birth, sex, and ethnicity.

[3]If these tests completed within 3 months of the screening visit, and the results are available, then these tests will not be required at this visit.

[4]Urine test and blood tests. Blood tests include sodium, potassium, chloride, bicarbonate, urea, serum creatinine, estimated glomerular filtration rate, AST, ALT, glucose, low density lipoprotein cholesterol, high density lipoprotein

cholesterol, total cholesterol, and triglycerides. Urinary analysis include hCG (for women only) and urinary albumin and creatinine for both men and women. For women, urine test is required to rule out pregnancy.

Laboratory assessments, concomitant medications, and medical history will be obtained from the electronic health record (EHR), which will be considered source for these assessments.

If a telehealth visit is conducted prior to randomization, then all information collected during these visits will be documented electronically, including the consent as outlined in the Consent Process section of the protocol. During the in-person screening and randomization visits, the electronic information will be verified and documented on paper CRFs. Any updates to previously reported information will be made at that point in time both electronically and if necessary, on paper versions of CRFs in accordance with Good Clinical Practice guidelines.

Other study-related data indicated in the visit schedule above will be collected via paper CRFs, which will serve as source documents. The data from the paper CRFs and EHR will be entered electronically into REDCap, housed at Northwestern University. See the appendix for current CRFs. We plan to use a "first / second pass" workflow, whereby one study team member will enter study data into the eCRFs and leave the data as "unverified". Then, a second study team member will review the data entered for accuracy and consistency in comparison to the EHR and paper CRFs. If there are any queries, then he / she will use the Data Resolution Workflow module in REDCap to resolve these issues. If there are no queries or data issues noted, then the second pass study team member will mark the form(s) as "complete". All study data must be entered into the electronic database within two business days after collection on paper CRFs. AEs may be deemed an exception to this rule as they have separate reporting requirements as outlined in the AE Reporting Plan and Data and Safety Monitoring Plan.

6. DATA AND SPECIMEN BANKING

Data will be collected on paper CRFs and entered into a REDCap database housed at Northwestern University within two business days of collection. The exception is the capture of AEs, which is specified in the AE Reporting Plan. Only study staff with Northwestern NetIDs will have access to the electronic database. We will restrict export rights to the study statistician only. All exports will be housed in secure Northwestern fsmresfiles folder directories with restricted access. These files are routinely backed up through Northwestern's Information Technology services. Refer to the DSMP and DMP for details on data and safety monitoring and quality checks. Blood and urine samples collected for this study will only be used for this study and will be destroyed after testing.

The results of this trial are not intended to support a New Drug Application (NDA) to bring the LDQT to market in the US. The study team will retain all study records required by applicable regulatory bodies in a secure and safe facility for a minimum period of seven years. Access to study records will be limited to study team members, unless through written application to and approval by the study Steering Committee.

7. SHARING RESULTS WITH PARTICIPANTS

When the study is over, research findings will be available at www.ClinicalTrials.gov (Identifier: NCT03640312), as required by US law. After all participants have completed the research study, each participant will receive a letter indicating which study drug he/she received when at randomization. The study staff will host an open forum at the Access Center for Discovery and Learning to share the study results. The study staff will be available to participants if they have questions regarding the results of the study. The research findings will also be published in the form of research articles or presented at scientific meetings. The sponsor, the National Heart, Lung, and Blood Institute of the NIH, requires that these research papers be made freely available to the public through PubMed Central (https://www.ncbi.nlm.nih.gov/pmc/) within 12 months of publication with data available thereafter in accordance with NHLBI policy through BioLINCC.

8. STUDY TIMELINES

An individual consenting participant will spend a maximum of 12 weeks (three months) participating in the QUARTET USA study. Refer to the study schedule above for details. We anticipate enrollment taking 30 months, assuming an average of 15 participants per month after an initial “ramp up” period. Thus, primary analyses will begin February 2022 with an estimated completion date of November 2022.

9. INCLUSION AND EXCLUSION CRITERIA

9.1. Inclusion Criteria:

- Adults (≥18 years).
- Spanish or English speaker.
- Previous documentation within the past 24 months of hypertension or high blood pressure (SBP 130-179 mmHg or DBP 80-109 mmHg) from general practitioner, pharmacist or health care professional (e.g., medical assistant, physician or nurse).

  And either:

  1) Untreated (automated) clinic SBP 140-179 or DBP 90-109 mmHg in the last 12 weeks.

  Or

  2) Monotherapy with clinic SBP 130-159 or DBP 85-99 mmHg in the last 12 weeks.

  A SBP or DBP level higher than these thresholds would lead to ineligibility. However, if an individual were eligible based on an in-range SBP or DBP level (i.e., isolated systolic

hypertension or isolated diastolic hypertension), then a lower corresponding DBP or SBP would not lead to exclusion.

And 1 of the following:

- Treatment naïve
- Currently not on treatment (not take in last 4 weeks)
- Currently taking 1 BP lowering drug (ACE, ARB, CCB, thiazide- or thiazide-like diuretic, BB, MRA, alpha blocker) at any dose

The blood pressure inclusion criteria align more closely with the QUARTET trial. The table shows the range of automated office (clinic) blood pressures for inclusion, as well as a comparison with the QUARTET study in Australia. Safety data from QUARTET (Australia) were published in August 2021.[10] Among 591 participants followed for 12 weeks, there were 7 (3%) SAEs in the intervention group (one each of positional vertigo, shortness of breath, non-cardiac chest pain, tonic clonic seizure, fracture of ankle, cholecystitis, and migraine) and 3 (1%) SAEs in the control group (one each of non-cardiac chest pain, pneumonia, and myocardial infarction). There were 12 (4.0%) treatment withdrawals for any event in the intervention group, versus seven (2.4%) in the control group.

	**SBP lower limit, mmHg**	**SBP upper limit, mmHg**	**DBP lower limit, mmHg**	**DBP upper limit, mmHg**
Automated office (clinic) measurement within the last 12 weeks, untreated	140	179	90	109
Automated office (clinic) measurement within the last 12 weeks, on monotherapy	130	159	85	99
Research grade blood pressure measurement in QUARTET Australia	No limit	No limit	No limit	No limit
Research grade blood pressure measurement (baseline mean)	115	None	60	None

A SBP or DBP level higher than these thresholds would lead to ineligibility. However, if an individual were eligible based on an in-range SBP or DBP level (i.e., isolated systolic hypertension or isolated diastolic hypertension), then a lower DBP or SBP would not lead to exclusion.

If either or both mean research grade blood pressures were lower than the listed SBP and DBP thresholds, then the participant would not be eligible.

9.2. Exclusion criteria:

- Known contraindication to candesartan, amlodipine, indapamide or bisoprolol.
- Previous diagnosis of coronary artery disease, stroke, or heart failure.
- Presence of significant proteinuria (based on 3+ proteinuria via spot urinalysis or >300 mg/dL of proteinuria based on random urinary albumin-to-creatinine ratio testing of 300 mg/g)
- Evidence of secondary cause of hypertension e.g., renal artery stenosis; significant renal impairment (eGFR <50 ml/min/1.73 m^2), raised serum potassium (above lab normal limit of 5.5 mEq/L).
- Women who are pregnant, breast feeding or of childbearing potential and are not using and do not plan to continue using medically acceptable form of contraception throughout the study (pharmacological or barrier methods).
- Concomitant illness, physical impairment or mental condition which in the opinion of the study team / primary care physician could interfere with the conduct of the study including outcome assessments.
- Participation in a concurrent interventional medical investigation or pharmacologic clinical trial. Patients in observational, natural history or epidemiological studies not involving an intervention are eligible.
- Participant's responsible primary care or other responsible physician believes it is not appropriate for participant to switch current monotherapy.
- Inability or unwillingness to provide written informed consent.
- Unable to complete study procedures.

This study will not include any of the following special populations: a) adults unable to consent; b) individuals who are not yet adults (minors): i.e. infants, children, or teenagers; c) pregnant women; or d) prisoners or other detained individuals.

We will identify a list of potentially eligible participants at the participating site(s) through the Epic-based health information technology system at Access Community Health Network. After obtaining a list of potential participants, the study staff will review these potential participants' charts in the EHR, ensure permission from the patients' treating physicians to contact for potential participation in the QUARTET USA study, and call or electronically message these potential participants to determine interest in an initial telephone screen. Electronic messages will be sent through MyChart. If patients express interest, then the study coordinator will schedule a two-day screening visit. During the screening visit, the coordinator will obtain informed consent and conduct baseline assessments with the study nurse to determine eligibility status. Potentially eligible participants will be fitted for an ABPM and asked to wear it for 24 hours and return to clinic. The study coordinator and nurse will conduct a final assessment on eligibility based on the 24-hour ABPM and any other outstanding assessments / results upon the participant's return to clinic. Eligible participants will then be randomized via the randomization module in REDCap. The randomization module will return a study drug kit number. This will preserve the blinding of treatment allocation, since only the study statistician (or representative as a backup) will have access to the master list of drug kit numbers and corresponding treatment codes.

10. PARTICIPANT POPULATION(S)

Accrual Number:	Category/Group: (Adults/Children Special/Vulnerable Populations)	Consented: Maximum Number to be Consented or Reviewed/Collected/Screened	Enrolled: Number to Complete the Study or Needed to Address the Research Question
Local	Adults	No Limit	87
Study-wide	Adults	No Limit	87
Total:	Adults	No Limit	87

11. RECRUITMENT METHODS

Participants will be recruited from two of the community clinics within Access Community Health Network using its Epic-based health information technology system. This system allows for rapid electronic identification of eligible participants based on routinely collected clinical data as well as direct communication with their clinicians to seek their permission to contact potential participants. This methodology has been used in numerous studies between Northwestern investigators and ACCESS for successful recruitment. Participants may be contacted for screening either by phone or through electronic messaging, through MyChart. After initial contact by phone or electronic messaging, participants may also be contacted via text for appointment scheduling and reminders and retention.

In the event of lagging recruitment or lack of diversity in study population to reach our targets, we plan to open up recruitment and enrollment efforts to additional Access Community Health Network site(s). If this occurs, then we will submit a protocol amendment and seek IRB approval prior to initiating study procedures at any additional sites.

12. COMPENSATION FOR PARTICIPATION IN RESEARCH ACTIVITIES

Study participants will be paid according to the following schedule via cash ($30 per study visit and reimbursement for transportation costs with CTA or PACE cards ($5 per round trip). The maximal amount to be paid for participating in this study is $115.

	Screening and Randomization		6-Week Follow-up Visit	12-week Follow-up Visit	
**Payment Schedule**	**Visit 1**	**Visit 2**	**Visit 3**	**Visit 4**	**Visit 5**
Incentive (cash)	$15 if ineligible to continue	$30	$30	-	$30

PACE, CTA, Lyft or equivalent	$5	$5	$5	$5	$5
Automated home blood pressure monitor		X			

Some participants may be ineligible after completing components of the screening and randomization visits. If the participant has attended a single visit and has been determined ineligible, they will be compensated $15. If the participant has attended two visits and has been determined ineligible or eligible, they will be compensated $30.

13. WITHDRAWAL OF PARTICIPANTS

If a participant is withdrawn or decides to withdraw from the study, then attempts will be made to conduct a close-out visit, which will include the assessments planned for the 12-week visit. Data that have been already collected will not be removed from the study database unless explicitly requested by the participant.

If the study physician / medical monitor / DSMB deems it unsafe for any participant to continue study drug or any study procedures, then he / she may withdraw the study participant without his / her consent. If a study participant experiences an AE requiring discontinuation of study drug, then we will collect any remaining study drug and refrain from any additional dispensation for that participant. We will continue to follow the participant and collect all required follow-up data per the study visit schedule. If a participant is incarcerated, becomes terminally ill, or experiences any other circumstance that would make continued involvement in the study unfeasible or unethical, then the study team may withdraw the participant without his / her consent. In all cases, any data already collected will not be removed from the study database and may still be used in analyses unless explicitly requested by the participant.

14. RISKS TO PARTICIPANTS

- The risks of taking a blood sample include pain, a bruise at the point where the blood is taken, redness and swelling of the vein, infection, and a rare risk of fainting.
- The ECG procedure may cause some redness or itching on the skin where the leads are placed.
- If participants are randomized to the control arm, then they will be taking candesartan, an FDA-approved drug for the treatment of hypertension (high blood pressure).
- If participants are randomized to the active intervention arm, then they will be taking the four-drug combination pill, which includes ultra-low doses of candesartan, amlodipine, indapamide, and bisoprolol. These drugs are each approved by the FDA for treatment of hypertension, but a combination pill of these medications at these lower doses has not been approved to date. The known side effects of each of these medicines are shown

below. These side effects typically go away after stopping the medication. If any of these side effects occur while a participant is in the trial, then they will be reported as AEs.

Very common: More than 1 out of 10 people have reported this side effect

Common: From 1 to 10 out of 100 people reported this side effect

Uncommon: From 1 to 10 out of 1,000 people have reported this side effect

Rare: From 1 to 10 out of 10,000 people have reported this side effect

Very rare: Less than 1 in 10,000 people have reported this side effect

The frequency of side effects in based on standard doses, and an important hypothesis of this study is that quarter doses, even in combination, will be better tolerated than a standard dose.

**Reported Side Effects**	**Amlodipine**	**Bisoprolol**	**Candesartan**	**Indapamide**
Allergic reactions like skin rash, itching or hives, or swelling of the face, lips, or tongue	Uncommon	Rare	Rare	Common
Anxiety		Uncommon		Uncommon
Blurred vision or changes in vision or hearing	Uncommon			Common
Breathing problems	Uncommon	Uncommon	Uncommon	
Change in sex drive or performance		Uncommon	Uncommon	
Chest pain	Rare	Common	Common	
Cold, tingling or numb hands or feet		Common		
Confusion		Uncommon		
Cough			Uncommon	
Depression		Uncommon		
Diarrhea		Common		Common
Dry mouth	Uncommon			Rare
Dry or burning eyes		Uncommon		
Facial flushing	Uncommon			

Feeling faint or lightheaded or falling			Very common	
Headache		Common	Common	Common
Infection or flu-like symptoms				Common
Irregular or fast heartbeat	Uncommon		Uncommon	Common
Irregular or slow heartbeat		Very common		Common
Loss of appetite				Common
Muscle aches and pains		Common		Common
Muscle cramps or spasms				Common
Nausea, vomiting	Uncommon	Common	Common	Uncommon
Passing less urine			Common	
Redness, blistering, peeling or loosening of the skin, including inside the mouth				Very rare
Stomach gas, pain	Common		Common	
Sweating		Uncommon		
Swelling of hands or feet			Uncommon	
Swelling of legs or ankles	Very common	Uncommon		
Tremors		Uncommon		
Trouble sleeping	Uncommon			
Unusually weak or tired	Uncommon			Common

- In addition to these risks, this research may harm participants in ways that are unknown. These may be a minor inconvenience or may be so severe as to cause death but this is not expected.
- This study involves the use of participants' identifiable, personal health information and there is a chance that a loss of confidentiality could occur. The researchers have procedures in place to lessen the possibility of this happening.
- The study drug may also hurt a pregnancy or fetus in ways that are unknown. These may be a minor inconvenience or may be so severe as to cause death. Participants currently pregnant or nursing will be excluded from this study, and eligibility is contingent on participants' willingness to use acceptable forms for birth control. If participants become pregnant during the course of the study, then they are asked to notify the study team, and the study team will halt study drug administration. The study team will

continue to follow these participants and collect any AE and study-related data through the final study time point.

15. POTENTIAL BENEFITS TO PARTICIPANTS

During this research study, participants will receive blood pressure lowering medication, which may help control their blood pressure during this period.

16. DATA MANAGEMENT AND CONFIDENTIALITY

Paper CRFs will be housed at the study site in a locked, secure location. Only authorized study staff, monitors or auditors will have access to these files. Electronic data will be stored in the eCRFs maintained using the Northwestern University REDCap platform. Thus, only those with an appropriate Northwestern NetID and password will have ability to enter and view study data. Export rights will be restricted to the study statistician only, and data will only be exported when necessary for reporting and quality control purposes to a restricted 'fsmresfiles' location at Northwestern. The randomization list and any blinded material will also only be restricted to the study statistician, with files located on fsmresfiles with restricted access.

To ensure data quality, we will build in field validation(s) and branching logic within the forms, use a first / second pass data entry workflow, and we will use the field comment logs and data resolution workflow module within REDCap. Before obtaining data entry rights in production, study team members must complete a training and pilot test entry of hypothetical data. We will document these training sessions and successful completion of any study data entry practicums.

Further, we will perform centralized monitoring through Data and Status Quality Reports (DSQRs) and REDCap reporting features. The DSQRs will use the REDCap application programming interface (API) functionality to export the study data, and then restructure and summarize the data using statistical software such as R or SAS. The output for these DSQRs will be housed on Northwestern University's secure servers with restricted access to study team members only. The reports will be reviewed and discussed weekly, on average, but once the systems are in place and code generated, the reports may be updated in real-time.

The contents of these reports may continually evolve throughout the course of the study, but they will focus on the following essential study data:

- Baseline information – screening and consent, enrollment, demographics
- Adherence to intervention (pill counts) and protocol adherence
- Primary and secondary response variables
- AEs / SAEs
- Concomitant medications
- Other pre-specified response variables and/or covariates (laboratory data, exploratory outcomes)

For the purposes of these reports, we will only present data in aggregate form (i.e., we will not summarize results by study arm) overall. In addition to these general data summarizations, we plan to employ statistical methods for monitoring clinical trial data.[14] The general strategies include:

- Exploration of:
    - Descriptive statistic summaries overall and by site for essential study data as indicated above.
    - Frequency of outlying values overall and by site.
    - Process measures overall and by site:
        - Screen failure rate
        - Outlier and inlier rates
        - Correlation coefficients of key covariates
        - Deviations and AEs by site
        - Timeliness of data entry
        - Proportion of dropouts / losses to follow-up
- Evaluating important response and safety data univariately and also longitudinal trajectories over time (within the same person).
- Multivariate techniques involving principal components (PC1 vs. PC2) and examining multivariate outliers. For example, we may examine mean for individual center vs. that of all other centers. Plot PCs for p-values for all variables and identify outliers (those outside of the central cloud).
- Tests on randomness – Benford's law on the distribution of digits.

16.1. Sample Size

A target of 87 participants will be randomized (1:1 allocation). The analytic sample size of 77 is anticipated based on 87 participants at randomization and a conservatively estimated 12% dropout rate by the 12-week follow-up time point based on 8% dropout rate observed through September 2021. This sample size is conservatively based on an independent two-sample t-test. The analysis methods, linear mixed models including a fixed baseline value term, will increase precision on intervention effect when controlling for relevant baseline covariates, thereby providing additional power of detecting intervention effect.

The initial, conservative sample size and power calculations based on an independent two-sample t-test suggested 80% power to detect a 5 mmHg difference in SBP between the intervention and comparator arms assuming a two-sided 5% level of significance and a 15 mmHg standard deviation in outcome. At the request of the DSMB, we conducted an interim conditional power analysis, taking into consideration information from both the QUARTET USA trial data as of August 2021 and further the QUARTET (Australia) results.[10] These interim analyses, incorporating information to date, suggested that a recruited sample size of at least 77, and a 12% dropout rate, would provide over 90% conditional power based on a sample of 87 randomized participants.

Previously, the protocol required the 24-hour ambulatory blood pressure assessments, and we thus conducted initial power calculations based on several *a priori* assumptions for this endpoint as follows: Since expected mean 24-hour ambulatory blood pressure may be more precise than office blood pressure (with a standard deviation of 12 mmHg vs. 15 mmHg for office blood pressure), over 95% power was anticipated with the original sample size (N=364) to detect a 5 mmHg difference across arms in this important secondary outcome under the same assumptions as outlined for our primary outcome (office blood pressure). However, subsequent protocol modifications allowed for optional 24-hour ambulatory blood pressure, and this outcome has been modified to become an exploratory outcome.

16.2. Statistical Analysis

The primary study analysis time point for all relevant outcomes is 12 weeks post randomization. The original analysis plan called for ANCOVA, controlling for baseline value of each relevant outcome in addition to the following baseline covariates: sex, age at baseline, race/ethnicity, health literacy level (indicator of limited literacy as defined by the Newest Vital Sign instrument), and an indicator of monotherapy at baseline (vs. untreated; this variable will serve as a stratification factor for randomization). These variables are deemed clinically relevant and important covariates; thus, all analyses will plan for adjustment for these covariates of interest (regardless of statistical significance in the current dataset) in predicting outcome in the present study. However, to better align with the analytic strategies of the QUARTET Australia study, and to make most efficient use of all follow-up data (both six-week and 12-week data), primary analyses will involve a linear mixed model with fixed study arm and baseline outcome value effects and a random participant effect to account for within-participant correlation. We plan to conduct both unadjusted (for potential covariates mentioned above) and adjusted analyses. The updated details of these analyses are reserved for the statistical analysis plan. The basic analytic model will be as follows for each outcome (*Y*) for participant *i (i=1…N)* at visit *j (j=1,2; corresponding to Week 6 and Week 12)*:

$$Y_{ij} = \beta_0 + \beta_1 \boldsymbol{I}(LDQT) + B_2(Y_{0i}) + B_3 visit + B_4(visit\ x\ study\ arm) + b_i + e_{ij}$$

Under the assumption that error terms $e_{ij} \sim N(0, \sigma^2)$ is the random error term, $b_i \sim N(0, \sigma_b^2)$ is the participant-level random effect. If visit-by-study arm (B_4) is insignificant at the 5% level, we will remove that term from the model, and subsequently, we will further examine for an overall main effect for visit (B_3). If insignificant, we will also remove that effect from the model and evaluate intervention effect via the primary hypothesis test of interest:

$$H_0: \beta_1 = 0\ vs. H_1: \beta_1 \neq 0$$

for each outcome. However, if the visit-by-study arm interaction term or the visit term alone is significant, we will plan to evaluate the 12-week contrast via model-estimated least squared means as our primary analyses. All between-arm differences at both six- and 12-week time points will be reported as model-based estimates, corresponding 95% confidence limits, and p-value of the corresponding hypothesis test.

Secondary analyses will utilize data from all time points via (generalized) linear mixed modeling (GLMM) methods with the following specifications: identity, logit, or log link for continuous, binary, or count outcomes, respectively; fixed arm, visit, visit-by-arm interaction, and aforementioned covariates; and random participant effect. For secondary analyses, the model adjusted Wald type III tests for fixed effects will first evaluate significance of a visit-by-arm interaction at the relaxed 10% level of significance. If insignificant at the 10% level, then this interaction term will be removed and the model Wald type III test for fixed arm effect will evaluate the overall intervention effect in this longitudinal model at the 10% level (since these analyses are exploratory and secondary).

Safety outcomes will be treated similarly: adverse event rates and relevant outcomes previous listed will be tabulated overall and by arm. Chi-squared tests or exact methods will evaluate the differences across arms in event rates at the participant level. For outcomes such as relevant side effects that may occur more than once in any participant, descriptive statistics or GLMMs as specified above will be examined, and continuous laboratory safety assessments will also be analyzed as specified above (GLMMs, as appropriate).

Procedures for control and handling of specimens are described within the Laboratory Procedures document.

17. PROVISIONS TO MONITOR THE DATA TO ENSURE THE SAFETY OF PARTICIPANTS

The DSMP documents the provisions to monitor the data and ensure safety of all study participants. Briefly, (1) we have convened an independent DSMB consisting of five members with diverse backgrounds and fields of expertise, including an expert in research methodology and ABPM, a biostatistician, a clinician-scientist with expertise in hypertension combination treatment trials and prevention of heart and kidney disease, an ethicist, and a patient representative; (2) the DSMB will meet at minimum every six months to review safety and essential study data; (3) we will employ centralized monitoring approaches as mentioned above to monitor data quality and safety; (4) we will program an email alert to study staff in the event of an SAE; (5) we plan for targeted on-site monitoring by an external clinical research associate; (6) and we will adhere to regulatory requirements for expedited AE / SAE reporting.

18. PROVISIONS TO PROTECT THE PRIVACY INTERESTS OF PARTICIPANTS

Information regarding patient privacy relevant to data and specimens is described within this protocol in section Data and Specimen Banking. All study related procedures and visits will occur at Access Community Health Network clinics which complies with federal regulations for patient privacy. All study-related visits will be completed in a designated enclosed study-room whereby only the study participant and study team (e.g. research coordinator and/or research nurse) will be present. The study room is in the back end of the health center far removed from clinical rooms, nurse stations, and reception/waiting room area whereby study participants may feel at ease to discuss matters as it relates to their participation in the research study.

19. COMPENSATION FOR RESEARCH-RELATED INJURY

This study is investigator-initiated. If participants become ill or are injured as a result of this study (medications, devices or procedures), they are directed to seek medical treatment through their doctor or treatment center of choice and to promptly tell the study doctor about any illness or injury. The researchers and clinical staff will not pay for medical care required because of a bad outcome resulting from participation in this research study. This does not keep participants from seeking to be paid back for care required because of a bad outcome.

20. ECONOMIC BURDEN TO PARTICIPANTS

We do not anticipate that patients will experience economic burdens related to participation in this study. Evaluation of hypertension will be performed according to clinical practice guidelines. Twelve-week treatment will be provided at no cost to participants. Participants will be reimbursed for their time and travel costs for study related visits.

21. CONSENT PROCESS

Participation will require informed consent from all participants. The study coordinator will consent participants at Access Community Health Network clinics or through a telehealth visit. Consent will be taken by the study coordinator and the research nurse will be available to consult during the consent process. Ample time will be devoted to the consent process according to participant request. Participants will be allowed time to consider the trial and ask questions and will be reminded they are free to leave the trial at any time. Prior to randomization, we will confirm participant consent and understanding of the trial. At each study follow-up visit, the consent will be confirmed.

- If conducted in person, both the participant and witness will sign and date two copies of the consent form if the participant agrees to the study procedures. A hard copy will be kept in the participant's file, housed at the health center where the study is being conducted (in a locked file cabinet in a locked closet). The participant will be provided with the other copy to take home.
- If conducted via a telehealth visit, the participant will have two options on how to complete consent form:
  - A. The participant will receive an electronic version of consent form (eConsent) as an automated survey sent from a 21 CFR, Part 11 compliant instance of REDCap. The participant will electronically sign and date the consent form, which will be stored in REDCap. The witness will electronically sign and date the consent form after the participant has completed the REDCap eConsent survey. A hard copy will be kept in the participant's file, housed at the health center where the study is being conducted (in a locked file cabinet in a locked closet). The participant will be sent a link to the executed electronic consent form.

- B. The participant will receive a hard copy of the consent form sent via regular mail or will print out a copy that is emailed to them through the patient's ACCESS MyChart account within Epic. The participant will provide a wet signature, will date the consent form, and will upload an image of the last page with their signature to their ACCESS MyChart account. A copy of the signed signature page of the consent will be saved in the patient's electronic health record. The witness will be able to print the signed form and provide a wet signature and date. A hard copy will be kept in the participant's file, housed at the health center where the study is being conducted (in a locked file cabinet in a locked closet).

22. PROTECTED HEALTH INFORMATION (PHI AND HIPAA)

This study involves collection and use of Protected Health Information. All participants will be identified by a unique study ID. Access to the full study database with identifying information (names, medical record numbers, dates, ages, email and telephone) will be restricted to authorized study personnel. Data collection will be secure, and these data will not be used for any other purpose than to contact the study participant during their participation in the trial and to disseminate results. Data shared from this study will be completely de-identified.

The informed consent form for this study includes HIPAA authorization.

23. NON-ENGLISH-SPEAKING PARTICIPANTS

We anticipate enrolling Spanish speaking participants in this study. Prior enrollment of any primarily Spanish speaking participants, all participant-facing study documents will be translated and back-translated for quality assurance, reviewed and approved by the IRB. All study drug will include labelling in both English and Spanish. Informed consent will be completed in either English or Spanish, according to participant preference.

24. QUALIFICATIONS TO CONDUCT RESEARCH AND RESOURCES AVAILABLE

For 25 years, Access Community Health Network (ACCESS) has been on the frontlines of community-based health care. ACCESS is the largest provider of primary care for Medicaid beneficiaries in Illinois and provides more primary care annually than the Cook County Health System. ACCESS provides a continuum of care model that connects patients to health care resources both within and beyond the walls of its 36 federally-qualified health centers (FQHCs). ACCESS's mission is to partner with patients at all points of care, providing high quality, patient centered health services accessible to all in their own communities. ACCESS is Joint Commission Accredited, and 35 of its health centers are Level III National Committee for Quality Assurance Patient Centered Medical Home Certified.

This study will enroll participants through the following centers:

- Martin T. Russo Family Health Center, at 245 S Gary Ave., Bloomingdale IL, 60108

- Ashland Family Health Center, at 5159 S. Ashland Ave., Chicago IL, 60609

ACCESS's patient demographics reflect the communities served: across all clinics, 40% are children age 17 years and under, 51% are Hispanic, 29% are African-American, 74% were at or below the 200% of the 2015 Federal Poverty Level ($23,540 annually), which is a common measure of economic stability.

The Russo and Ashland clinics serve over 2000 patients per month. Additional centers will be contacted via email, text message, or phone call using a MyChart-based communication system (Epic campaigns). Additional sites can also brought on based on the patient enrollment. At minimum, hall-time research nurses and project coordinators will support QUARTET USA at each location for the duration of the trial. All study-related visits will occur with the research nurse and coordinator. Co-investigator Dr. Jairo Mejia serves as the Chief Medical Officer for ACCESS and is based at the Russo clinic as the site principal investigator. Dr. Daneen Woodard is based at the Ashland clinic as the site principal investigator.

The QUARTET USA team holds routine meetings on a weekly basis with all operations staff. All members of the QUARTET USA team have previous experience in clinical trials and have current certification in Protection of Human Subjects and Good Clinical Practice.

25. MULTI-SITE OR COLLABORATIVE RESEARCH:

Northwestern investigators will collaborate with the research team at Washington University in St. Louis and Access Community Health Network. Study procedures will occur at the Access Community Health Network, where informed consent will be sought and study procedures will be conducted. The Northwestern IRB will serve as the IRB of record, and no activities will occur at Washington University in St. Louis until the SMART IRB LOA reliance agreement has been fully executed and appropriate local regulatory processes are followed and completed. The project is federally funded. The IRB approved the initial protocol before the Cooperative Research Requirement effective date. A single IRB is not required but is permissible. Northwestern University has served as the prime institution since the study start in 2018. As of January 2022, the prime institution will be Washington University in St. Louis based on the institutional change by the contact mPI, Dr. Mark Huffman. Enrollment will be completed in May 2022. The Access Community Health Network has agreed for Northwestern to serve as the IRB of record in accordance with their local policies.

REFERENCES

1. GBD 2013 Mortality and Causes of Death Collaborators. Global, regional, and national age–sex specific all-cause and cause-specific mortality for 240 causes of death, 1990–2013: a systematic analysis for the Global Burden of Disease Study 2013. *Lancet*. 2015;385:117–171.

2. Muntner P, Carey RM, Gidding S, Jones DW, Taler SJ, Wright JT Jr, Whelton PK. Potential U.S. population impact of the 2017 ACC/AHA High Blood Pressure Guideline. *J Am Coll Cardiol*. 2018 Jan 16;71(2):109-118.

3. Whelton PK, Carey RM, Aronow WS, Casey DE, Collins KJ, Dennison-Himmelfarb C, DePalma SM, Gidding S, Jamerson KA, Jones DW, MacLaughlin EJ, Muntner P, Ovbiagele B, Smith SC, Spencer CC, Stafford RS, Taler SJ, Thomas RJ, Williams KA, Williamson JD, Wright JT. 2017 ACC/AHA/AAPA/ABC/ACPM/AGS/APhA/ASH/ASPC/NMA/PCNA Guideline for the prevention, detection, evaluation, and management of high blood pressure in adults: a report of the American College of Cardiology/American Heart Association Task Force on Clinical Practice Guidelines. *J Am Coll Cardiol*. 2017; Nov 7. pii: S0735-1097(17)41519-1.

4. The SPRINT Research Group. A randomized trial of intensive versus standard blood-pressure control. *N Engl J Med*. 2015;373:2103–2116.

5. Wald DS, Law M, Morris JK, Bestwick JP, Wald NJ. Combination therapy versus monotherapy in reducing blood pressure: meta-analysis on 11,000 participants from 42 trials. *Am J Med*. 2009 Mar;122(3):290-300.

6. Bangalore S, Kamalakkannan G, Parkar S, Messerli FH. Fixed-dose combinations improve medication compliance: a meta-analysis. *Am J Med*. 2007;120:713–719.

7. Sundström J, Arima H, Jackson R, Turnbull F, Rahimi K, Chalmers J, Woodward M, Neal B. Effects of blood pressure reduction in mild hypertension. *Ann Intern Med*. 2015;162:184.

8. Chalmers J, Arima H, Harrap S, Touyz RM, Park JB. Global survey of current practice in management of hypertension as reported by societies affiliated with the international society of hypertension. *J Hypertension*. 2013;31:1043–1048.

9. Webster R, Salam A, de Silva HA, Selak V, Stepien S, Rajapakse S, Amarasekara S, Amarasena N, Billot L, de Silva AP, Fernando M, Guggilla R, Jan S, Jayawardena J, Maulik PK, Mendis S, Mendis S, Munasinghe J, Naik N, Prabhakaran D, Ranasinghe G, Thom S, Tisserra N, Senaratne V, Wijekoon S, Wijeyasingam S, Rodgers A, Patel A; TRIUMPH Study Group. Fixed low-dose triple combination antihypertensive medication vs usual care for blood pressure control in patients with mild to moderate hypertension in Sri Lanka: A randomized clinical trial. *JAMA*. 2018 Aug 14;320(6):566-579.12.

10. Chow CK, Atkins ER, Hillis GS, Nelson MR, Reid CM, Schlaich MP, Hay P, Rogers K, Billot L, Burke M, Chalmers J, Neal B, Patel A, Usherwood T, Webster R, Rodgers A; QUARTET Investigators. Initial treatment with a single pill containing quadruple

combination of quarter doses of blood pressure medicines versus standard dose monotherapy in patients with hypertension (QUARTET): a phase 3, randomised, double-blind, active-controlled trial. Lancet. 2021 Aug 27:S0140-6736(21)01922-X. doi: 10.1016/S0140-6736(21)01922-X. Epub ahead of print. PMID: 34469767.

11. O'Brien E, Parati G, Stergiou G, Asmar R, Beilin L, Bilo G, Clement D, de la Sierra A, de Leeuw P, Dolan E, Fagard R, Graves J, Head GA, Imai Y, Kario K, Lurbe E, Mallion JM, Mancia G, Mengden T, Myers M, Ogedegbe G, Ohkubo T, Omboni S, Palatini P, Redon J, Ruilope LM, Shennan A, Staessen JA, vanMontfrans G, Verdecchia P, Waeber B, Wang J, Zanchetti A, Zhang Y; European Society of Hypertension Working Group on Blood Pressure Monitoring. European Society of Hypertension position paper on ambulatory blood pressure monitoring. *J Hypertens*. 2013 Sep;31(9):1731-68.

12. Muntner P, Lewis CE, Diaz KM, Carson AP, Kim Y, Calhoun D, Yano Y, Viera AJ, Shimbo D. Racial differences in abnormal ambulatory blood pressure monitoring measures: Results from the Coronary Artery Risk Development in Young Adults (CARDIA) study. *Am J Hypertens*. 2015 May;28(5):640-8.

13. Jamerson K, Weber MA, Bakris GL, Dahlöf B, Pitt B, Shi V, Hester A, Gupte J, Gatlin M, Velazquez EJ; ACCOMPLISH Trial Investigators. Benazepril plus amlodipine or hydrochlorothiazide for hypertension in high-risk patients. *N Engl J Med*. 2008 Dec 4;359(23):2417-28.

14. Kirkwood AA, Cox T, Hackshaw A. Application of methods for central statistical monitoring in clinical trials. *Clin Trials*. 2013 Oct;10(5):783-806.
