# Supplementary material for: Efficacy and safety of a four-drug, quarter-dose treatment for hypertension: the QUARTET USA randomized trial
Source: Hypertens Res. 2024 Apr 8;47(6):1668–77. doi: 10.1038/s41440-024-01658-y (PMC11150153; doi:10.1038/s41440-024-01658-y)
Supplement: Supplementary file 3 — Statistical Analysis Plan [file 41440_2024_1658_MOESM3_ESM.pdf]

STATISTICAL ANALYSIS PLAN (SAP)

QUARTET USA:
A double blind randomized controlled trial to assess the efficacy and safety of a quadruple ultra-low-dose treatment for hypertension

February 9, 2022

Version 2.0

IRB Number: STU00205834

List of Abbreviations

ABPM	Ambulatory blood pressure monitoring
ACE-I	Angiotensin-converting-enzyme inhibitor
AE	Adverse event
ALP	Alkaline phosphatase, a liver function test
ALT	Alanine transaminase, a liver function test
ANCOVA	Analysis of covariance
API	Application Programming Interface
ARB	Angiotensin II receptor blocker
AST	Aspartate Aminotransferase, a liver function test
BB	Beta blocker
BMI	Body mass index
BP	Blood pressure
CABG	Coronary artery bypass graft, a type of surgery that improves blood flow to the heart
CAD	Coronary artery disease
CCB	Calcium channel blocker
CRF	Case report form
DBP	Diastolic blood pressure
DMP	Data Management Plan
DSMB	Data and Safety Monitoring Board
DSMP	Data and Safety Monitoring Plan
ECG	Electrocardiogram
ECT	Electroconvulsive therapy, a medical treatment used in patients with severe, major depression
eGFR	Estimated glomerular filtration rate, which estimates kidney function
HER	Electronic health record
GCP	Good clinical practice
GGT	Gamma-glutamyl transferase, a liver function test
HDL-c	High density lipoprotein cholesterol
IPD	Individual participant data
mITT	(modified) Intention-to-treat
LDQT	(ultra-) Low-dose quadruple combination therapy
LVH	Left ventricular hypertrophy, or muscle thickening of the heart's left pumping chamber
MI	Myocardial infarction, also known as a heart attack
MOP	Manual of Operating Procedures
PCI	Percutaneous coronary intervention, a non-surgical procedure to open narrowings or blockages of the coronary arteries
PROMIS	Patient-Reported Outcome Measurement Information System
QRS	An ECG-derived interval that represents ventricular depolarization
QTc	An ECG-derived interval that represents the time from ventricular depolarization to repolarization
REDCap	Research Electronic Data Capture
SAE	Serious adverse event
SAP	Statistical Analysis Plan

SBP	Systolic blood pressure
TIA	Transit ischemic attack, a stroke-like attack resolves within 24 hours
UACR	Urine albumin: creatinine ratio

STATISTICAL ANALYSIS PLAN (SAP)

QUARTET USA:
A double blind, randomized controlled trial to assess the efficacy and safety of a quadruple ultra-low-dose treatment for hypertension

Co-Principal Investigator: Mark D. Huffman, MD, MPH
Co-Principal Investigator: Jody D. Ciolino, PhD

1. INTRODUCTION

This document outlines the proposed analyses for the QUARTET USA phase II clinical trial, which aims to compare clinical and safety outcomes for adult participants with elevated blood pressure (BP) at baseline who receive treatment with ultra-low-dose quadruple-combination therapy (LDQT) to those outcomes in participants given standard dose monotherapy at 12 weeks. Thus, we plan to conduct a two-arm, double-blind randomized controlled trial with equal allocation (1:1) in adults with uncontrolled blood pressure who are eligible for monotherapy. The purpose of this document is to provide detail regarding the statistical analysis plan (SAP) for this study.

<u>Study Aims</u>
The overarching study aims are as follows:

Aim 1: To investigate whether initiating treatment with ultra-low-dose quadruple-combination therapy ("LDQT") including candesartan 2mg, amlodipine 1.25mg, indapamide 0.625mg, and bisoprolol 2.5mg will lower office blood pressure at 12 weeks more effectively, and with no increase in side effects, compared to initiating standard dose monotherapy (candesartan 8mg) in adults with elevated blood pressure who are eligible for monotherapy based on the 2017 AHA/ACC guideline.

We hypothesize that initiating treatment with LDQT will lower office blood pressure at 12 weeks more effectively, and with no increase in side effects, compared to initiating standard dose monotherapy in adults with elevated blood pressure who are eligible for monotherapy.

Aim 2: To investigate whether initiating treatment with LDQT will lower mean 24-hour ambulatory blood pressure at 12 weeks more effectively, and with no increase in side effects, compared to initiating standard dose monotherapy in adults with elevated blood pressure who are eligible for monotherapy.

We hypothesize that initiating treatment with LDQT will lower mean 24-hour ambulatory blood pressure at 12 weeks more effectively, and with no increase in side effects, compared to initiating standard dose monotherapy in adults with elevated blood pressure who are eligible for monotherapy.

Exploratory Aim 1: Assess heterogeneity of treatment effect by hypothesized moderators (age, sex, race/ethnicity, and health literacy level).
We hypothesize that the treatment effect of LDQT will be greater in participants with limited health literacy than those with adequate health literacy. We also hypothesize that treatment effect of LDQT will differ by age, sex, and race/ethnicity subgroups.

Exploratory Aim 2: To evaluate acceptability, preferences, and lessons for implementation of LDQT among patients and clinicians using mixed methods.

We hypothesize that patients and clinicians will prefer LDQT more than standard dose monotherapy for initial blood pressure lowering therapy; we further hypothesize that LDQT will be simpler and easier for both patients and clinicians than standard dose monotherapy for initial blood pressure lowering therapy, including patients with low health literacy.

This SAP will focus on the details of analyses for Aims 1, 2, and part of exploratory Aim 1 (pertaining to age, sex, and race/ethnicity as potential moderators); we reserve details of the exploration of health literacy and implementation analyses (exploratory Aim 2) for a separate document.

Study time points include baseline assessment (completed over approximately two days), a six-week follow-up time point, and a 12-week follow-up time point (also completed over approximately two days).

2. STUDY OUTCOMES

In the sections below, we include the relevant specific field names for variables within the study database as of the time of SAP creation. These are indicated by the [brackets].

<u>Primary Outcome</u>
Primary efficacy outcome is **mean change (from baseline) in automated office systolic blood pressure (SBP) at 12 weeks** [sbpavg], and analyses will compare this change across arms for primary outcome analyses, adjusting for baseline.

<u>Secondary Outcomes</u>
Secondary efficacy outcomes include:

1) Mean automated office SBP at 12 weeks, adjusted for baseline values [sbpavg].
2) **Other BP measures** that will be compared across arms, controlling for baseline values:
    a. Mean change (from baseline) in automated office SBP [sbpavg] at six weeks.
    b. Mean automated office SBP [sbpavg] at six weeks.
    c. Mean change (from baseline) in automated office DBP [dbpavg] at six and 12 weeks.
    d. Mean automated office DBP [dbpavg] at six and12 weeks.
    e. Proportion of patients with hypertension control (SBP < 130 mmHg and DBP <80 mmHg) at six and 12 weeks [sbpavg, dbpavg].
    f. Proportion of patients requiring step-up treatment [amlo_dispnd].
    g. Proportion of patients with adverse event-free hypertension control (SBP < 130 mmHg and DBP < 80 mmHg [sbpavg, dbpavg]). For the purposes of this outcome, we will define adverse event-free as absence of any events that are documented as either possibly, probably, or definitely related to study medication ([saerelate] = 3, 4, 5).
3) **Medication adherence:** proportion of participants deemed adherent to study medication based on the 80% criterion (i.e., participant took at least 80% of assigned study medication). This information will be captured via:
    a. Objective pill counts [capsret6, lost6, capsret12, lost12], and
    b. Self-reported measures [forget, miss30, miss7] in addition to the objective pill counts.
4) **Health-related quality of life:** Mean change from baseline in health-related quality of life

using PROMIS Global Health instrument [global01—global10]. There are two domains within this construct:

a. Global Mental Health (assessed via a T-score)
b. Global Physical Health (assessed via a T-score)

Safety Outcomes

We plan to evaluate the following safety outcomes:

1) Proportion of participants with *any* SAE according to the Good Clinical Practice (GCP) definition [sae_present, saedeath, saelifethrt, saehosp, saedisp, saecong, saeimpevnt].
2) Proportion of participants with *any* potentially relevant side effect ([sae_term] refer to the adverse event case report form and the list of relevant side effects from the informed consent form).
3) Rate of relevant side effects at the participant level (i.e., count per participant [sae_term]).
4) Mean change (from baseline) in continuous serum potassium (mEq/L), controlling for baseline value [potassium].
5) Mean change (from baseline) in continuous serum sodium (mEq/L), controlling for baseline value [sodium].
6) Mean change (from baseline) in continuous blood urea nitrogen (mg/dl), controlling for baseline value [urea].
7) Mean change (from baseline) in continuous serum creatinine (mg/dl), controlling for baseline value [creat].

Exploratory Outcomes

The following outcomes are relevant to the overall aims; however, they carry less weight, and we consider them exploratory in nature. This SAP will not focus in detail on analyses of these outcomes, but we anticipate the general analytic approach to apply. In the event of small numbers that would make modeling infeasible, these data may be reported as descriptive statistics.

ABPM measures

24-hour ambulatory blood pressure monitor (ABPM) measures at 12 weeks adjusted for baseline values:

1) Mean 24-hour SBP and DBP [overall_abpsbp, overall_abpdpb].
2) Mean daytime (0600 to 2200) [awake_abpsbp, awake_abpdbp] SBP and DBP.
3) Mean nighttime (2200 to 0600) SBP and DBP [asleep_abpsbp, asleep_abpdbp].
4) Proportion of dippers [sys_dip, dia_dip], defined as nighttime BP falling more than 10% from the daytime values OR night / day blood pressure ratio less than 0.9 and greater than 0.8 with normal diurnal blood pressure pattern.
5) Mean daytime SBP and DBP load. Load is defined as the percentage of abnormally elevated readings; daytime SBP / DBP elevated readings would be 130 / 80 mmHg or above.
6) Mean nighttime SBP and DBP load. Abnormally elevated readings for nighttime SBP / DBP would be 120 / 70 mmHg or above.
7) Percentage of participants with morning surge, calculated as the difference between the mean SBP during the morning hours and nighttime trough SBP. Trough SBP is defined as the mean of three SBP measurements: the lowest nighttime SBP and the measurements immediately preceding and following this measurement.
8) Coefficient of variation of SBP and DBP assessed through 24-hour ambulatory blood pressure as defined as the ratio of the 24-hour standard deviation of BP / mean 24-hour value.

9) Day-night variability (SDdn), which uses the standard deviation (SD) for daytime measurements and, separately for nighttime measurements, to calculate a weighted mean of these SDs.
10) Average real variability (ARV), calculated as the average absolute difference between consecutive readings over the 24-hour ABPM period.

We will assess additional exploratory outcomes as needed to inform individual participant data (IPD) meta-analysis with international QUARTET studies. The details of these IPD analyses are reserved for a separate analytic plan.

3. DEMOGRAPHICS AND BASELINE ASSESSMENTS

The following are specific demographic / baseline assessments of interest for analyses. Primary analyses will adjust for these covariates as we anticipate they will influence outcome. We plan to report both model-adjusted and simple unadjusted intervention effect estimates. In the cases of adjusted models, we will include the following variables as fixed effects, regardless of significance:

1) Sex [sex].
2) Age [age].
3) Race/ethnicity [ethnic]. We plan to categorize participants into White (ethnic = 1), Hispanic (ethnic = 3, 4, 5, 6), African American (ethnic = 2), or other categories. In the event of low cell counts in any one category, we may consider collapsing categories, foregoing adjustment for race (if collapsing cannot be justified scientifically), or failing to adjust for race altogether. We may also consider another potential covariate that is heavily related to race and ethnicity or conduct sensitivity analyses under different parameterizations / assumptions.
4) Literacy score according to the Newest Vital Sign (NVS, sum of [vs_totcal, vs_carb, vs_fat, vs_daily, vs_safe, vs_peanut]) as defined per scoring manual. The score ranges from 0-6, with 0-1 indicating limited literacy, 2-3 indicating likely limited literacy, and 4-6 indicating adequate literacy.
5) Indicator of monotherapy at baseline [inc04 = 3].

Additional demographics in general include:

1) Country of birth [birth].
2) Insurance status [insurance].
3) Education [edu].
4) Employment status [emplst].
5) Income [income_usd].
6) Marital status [marst].
7) Number of people in household [pple].

Note that some additional exploratory analyses may examine these additional demographic variables as covariates and/or effect modifiers as well. We will label any exploratory analyses involving additional potential covariates as post hoc in any dissemination materials.

4. DATA STORAGE

Data will be collected and managed using Research Electronic Data Capture (REDCap) housed at Northwestern University's Clinical and Translational Sciences Institute (CTSA), NUCATS [1]. REDCap is a secure, web-based application designed for research studies that provides an intuitive interface for validated data entry, audit trails for tracking data manipulation and export procedures,

and automated export procedures for seamless data downloads to common statistical packages, and procedures for importing data from external sources. Refer to the study Data Management Plan (DMP) for details.

5. RANDOMIZATION METHODS

We plan for equal allocation (1:1) across study arms; the study statistician (Co-PI: Ciolino) generated a randomization list using random block assignments (i.e., randomly varying block sizes). The details of the block sizes and number of blocks will remain confidential until study completion. The study statistician uploaded a “Development” randomization list and a separate “Production” randomization list into REDCap. Each participant will be assigned a randomly-generated kit number that will correspond to either an active comparator (candesartan) or investigational product (LDQT) drug kit. The randomization lists are housed on Northwestern University’s “FSMResFiles” with restricted access such that only unblinded individuals can access. Neither the study coordinator / study nurse / individual assigning the study kit numbers nor the participants will have ability to determine which kit numbers correspond to each arm as they were generated via a random uniform distribution with the seed number, block sizes, and subsequently sorted randomization lists restricted to this set of folders on Northwestern University’s servers. Randomization does not involve any stratification factors; however, with the addition of a second study site, the randomization is de facto stratified by site (i.e., each site has its own randomization sequence).

6. STATISTICAL METHODS

Descriptive statistics will summarize participant demographics and baseline clinical outcomes overall and across arms: proportion (percentages) for categorical variables; mean (± standard deviation) for continuous variables; and median (interquartile range) for skewed or count variables. Analyses in general will employ normal theory methods and residual diagnostics will evaluate validity of assumptions; where appropriate (i.e., in the event of low cell counts for categorical data or questions of normality), transformation of variables, nonparametric methods, or exact tests may be employed. All primary efficacy and safety analyses will be pre-specified as outlined in this SAP, and deviations from planned analyses or post hoc analyses will be labeled as such in any reports or dissemination materials.

Analyses will assume a two-sided 5% type I error rate unless otherwise specified; there will be some exploratory analyses that will involve a relaxed type I error rate (10%). There will be no corrections made for multiple hypothesis tests, as this is a phase II study evaluating preliminary efficacy.

<u>Planned Primary, Secondary, Safety Analyses</u>
The primary study analysis time point for all relevant outcomes is 12 weeks post randomization. The original analysis plan called for an analysis of covariance (ANCOVA), controlling for baseline value of each relevant outcome in addition to the following baseline covariates: sex, age at baseline, race/ethnicity, health literacy level (indicator of limited literacy as defined by the Newest Vital Sign instrument), and an indicator of monotherapy at baseline (vs. untreated). We deem these variables clinically relevant, important covariates; thus, all analyses will plan for adjustment for these covariates of interest (regardless of statistical significance in the current dataset) in evaluating efficacy of intervention in the present study. However, to better align with the analytic strategies of the QUARTET Australia study, and to make most efficient use of all follow-up data (both six-week and 12-week data), primary analyses will involve a linear mixed model with fixed study arm and baseline outcome value effects and a random participant effect to account for within-participant correlation. We

plan to conduct both unadjusted (for potential covariates mentioned above) and adjusted analyses. The updated details of these analyses are reserved for the statistical analysis plan. The basic analytic model will be as follows for each outcome (*Y*) for participant *i (i=1…N)* at visit *j (j=1,2; corresponding to Week 6 and Week 12)*:

$$Y_{ij} = \beta_0 + \beta_1 \boldsymbol{I}(LDQT) + B_2(Y_{0i}) + B_3 visit + B_4(visit\ x\ study\ arm) + b_i + e_{ij}$$

Under the assumption that error terms $e_{ij} \sim N(0, \sigma^2)$ is the random error term, $b_i \sim N(0, \sigma_b^2)$ is the participant-level random effect. If visit-by-study arm (B_4) is insignificant at the 5% level, we will remove that term from the model, and subsequently, we will further examine for an overall main effect for visit (B_3). If insignificant, we will also remove that effect from the model and evaluate intervention effect via the primary hypothesis test of interest:

$$H_0: \beta_1 = 0\ vs. H_1: \beta_1 \neq 0$$

for each outcome. However, if the visit-by-study arm interaction term or the visit term alone is significant, we will plan to evaluate the 12-week contrast via model-estimated least squared means as our primary analyses. All between-arm differences at both six- and 12-week time points will be reported as model-based estimates, corresponding 95% confidence limits, and p-value of the corresponding hypothesis test.

Secondary analyses (i.e., those for the secondary and exploratory outcomes of interest) will utilize data from all time points via (generalized) linear mixed modeling (GLMM) methods with the following specifications: identity, logit, or log link for continuous, binary, or count outcomes, respectively; fixed arm, visit, visit-by-arm interaction, and aforementioned covariates; and random participant effect. For secondary analyses, the model adjusted Wald type III tests for fixed effects will first evaluate significance of a visit-by-arm interaction at the 5% level of significance. If insignificant at the 5% level, then this interaction term will be removed and the model Wald type III test for fixed arm effect will evaluate the overall intervention effect in this longitudinal model at the 5% level.

The table below summarizes the general modeling strategy for each outcome. In each case, we plan to conduct both adjusted and unadjusted analyses. Adjusted analyses will include the aforementioned covariates.

Table: Modeling Strategy by Outcome

Outcome	Variable Type	Model Link Assumption	Note on Covariates
Primary, secondary / exploratory blood pressure measurements at individual study time points	Continuous (assume normality)	Identity	Same as in primary outcome analyses
Dipping at 12 weeks	Binary / categorical	Logit	Indicator of dipping status at baseline in addition to other covariates as in primary analyses
Hypertension control at individual study time points	Binary / categorical	Logit	Same as in primary outcome analyses, including baseline SBP
Requirement for amlodipine add-on therapy	Binary / categorical	Logit	Same as in primary outcome analyses, including baseline SBP
Adverse event-free hypertension control at each study time point	Binary / categorical	Logit	Same as in primary outcome analyses, including baseline SBP
Medication adherence (at least 80%)	Binary / categorical	Logit	Same as in primary outcome analyses, including baseline SBP
Change in PROMIS Global Health	Continuous (assume normality)	Identity	Same as in primary outcome analyses
Indicator for *any* SAE	Binary / categorical	Logit	Same as in primary outcome analyses, including baseline SBP
Indicator for *any* relevant side effect	Binary / categorical	Logit	Same as in primary outcome analyses, including baseline SBP
Rate of relevant side effects	Count (Poisson assumption)	Log	Same as in primary outcome analyses, including baseline SBP
Continuous laboratory measurements (potassium, sodium, urea, creatinine)	Continuous (assume normality)	Identity	Same as in primary outcome analyses

Subgroup Analyses and Heterogeneity of Intervention Effects

An exploratory objective of this study is to assess heterogeneity of treatment effect by the following potential hypothesized moderators: age, sex, and race/ethnicity. To address this, we will analyze primary and secondary blood pressure (12-week SBP, DBP) outcomes via the aforementioned

LMMs; we will add an arm-by-<potential moderator> effect in the original model specified above for each potential moderator of interest. Moderation effects will be explored via model-adjusted type III Wald test for fixed effects at the relaxed 10% level of significance. If significant, then we will examine the intervention effect within each subgroup via a series LMMs: male/female, age category, racial/ethnic category.

Agreement between Outcomes

We will use simple sample Pearson correlation coefficients (and 95% confidence limits), Bland-Altman plots, or both to examine agreement among continuous outcome measures (primarily focused on office SBP and 24-hour SBP measures). Though these analyses are not the primary focus of this trial and its results, we will use agreement analyses to make inference regarding quality of SBP measurement methods and variability.

Analyses Contingent on Add-on Therapy Requirements

If there is evidence of a difference in proportion of participants requiring amlodipine add-on therapy at six weeks across arms, then we will also explore dividing the sample into four strata: (1) those that required amlodipine add-on treatment + received active control, (2) those that required add-on + received LDQT, (3) those that did not require add-on + received active control, and (4) those that did not require add-on and received LDQT. Depending on cell counts, we will attempt a series of exploratory analyses for key outcomes (SBP, DBP) to evaluate an effect. Additional analyses of this nature will be indicated as exploratory.

7. ANALYTIC DATASET

Analyses will include the (modified) intention-to-treat (mITT) dataset, whereby all those participants with data at any follow-up time point and baseline to contribute to analyses will be included in analyses according to arm to which they were randomized, regardless of adherence to the study protocol. We will conduct a sensitivity analysis on the per protocol dataset (defined as 80% treatment regimen adherence) since precise estimates of intervention effect (if any) on outcomes are important in a phase II study.

Power and sample size considerations allowed for some missing data (20%); however, in the event of large amounts of missing data (i.e., more than 10%), multiple imputation analyses will be explored. We will examine rates of missing data for all variables and determine whether the rates vary by participant characteristics, etc. These summarizations will inform potential biases resulting from missing data. Mixed effects models planned for longitudinal analysis are generally robust for unbalanced data across study time points. Additional sensitivity analyses will explore multiple imputation methods and the global sensitivity analysis to evaluate overall trial robustness [2]. These analyses will again serve as sensitivity analyses to the previously outlined analyses.

8. POWER AND SAMPLE SIZE CONSIDERATIONS

The initial sample size calculations called for a total of 365 participants to be randomized (1:1 allocation). We anticipated an analytic sample size of 292 based on 365 participants at randomization and a 20% dropout rate by the 12-week follow-up time point. We originally based sample size and power calculations conservatively on an independent two-sample t-test. Based on results of interim analyses (refer to Section 10 for details), we updated our recruitment target to 87 participants (1:1 allocation). The analytic sample size of 77 is anticipated based on 87 participants at randomization

and a conservatively estimated 12% dropout rate by the 12-week follow-up time point based on 8% dropout rate observed through September 2021.

The initial, conservative plan for primary outcome analyses involving an independent two-sample t-test provided an estimated 80% power to detect a 5 mmHg difference in SBP between the intervention and comparator arms assuming a two-sided 5% level of significance and a 15 mmHg standard deviation in outcome. This estimate is based on a 2017 Cochrane systematic review update evaluating the effects of fixed-dose combination therapy and systematic review on quarter dose combination therapy, and a pilot trial of quarter-dose combination therapy [3]. We assumed baseline SBP has a moderate correlation with follow-up SBP ($r \approx 0.50$-0.6); under this assumption, sample size calculations based on ANCOVA has the potential to allow for over 90% power under the same assumptions for remaining parameters.

At the request of the DSMB, we conducted an interim conditional power analysis, taking into consideration information from both the QUARTET USA trial data as of August 2021 and further the QUARTET (Australia) results.[10] These interim analyses, incorporating information to date, suggested that a recruited sample size of at least 77, and a 12% dropout rate, would provide over 90% conditional power based on a sample of 87 randomized participants.

Previously, the protocol required the 24-hour ambulatory blood pressure assessments, and we thus conducted initial power calculations based on several a priori assumptions for this endpoint as follows: Since expected mean 24-hour ambulatory blood pressure may be more precise than office blood pressure (with a standard deviation of 12 mmHg vs. 15 mmHg for office blood pressure), we estimate over 95% power with the planned sample size to detect a 5 mmHg difference across arms in this important secondary outcome, under the same assumptions as outlined for our primary outcome (office blood pressure). However, subsequent protocol modifications allowed for optional 24-hour ambulatory blood pressure, and this outcome has been modified to become an exploratory outcome.

9. TECHNICAL DETAILS

The SAP is subject to version control, and we anticipate modifications to analytic plans be documented herein. As in any study, the analytic plan may change due to assumption violations, logistical issues, unexpected empirical distributions of study outcomes, or a combination thereof. In these cases, the SAP will be updated accordingly. All analyses will be performed via SAS version 9.4 or higher (The SAS Institute; Cary, NC) or R version 3.6.0 or higher (The R Foundation for Statistical Computing platform). Table and figure formatting and style may be dictated by mode of dissemination or specific target journal(s) for results dissemination.

Summary of updates:
Version 2.0:

1) Removed reference to heterogeneity of treatment effects based on health literacy as these analyses will be outlined in a separate document.
2) Moved ABPM to exploratory analyses.
3) Updated target sample size based on interim analyses at request of the DSMB.
4) Updated modeling strategy overall to involve mixed modelling techniques (previously planned for ANCOVA at Week 12, only) to use both Week 6 and Week 12 data in analysis models. All longitudinal models will first explore a time-by-arm interaction term at the 5% level of significance in evaluating treatment effect.
5) Specified minor details on treatment of covariates in analyses.

6) Added in plans to explore potential strata combining study arm and add-on therapy.

10. TIMELINE FOR ANALYSES

As this is a phase II clinical trial, the original analysis plan did not include any formal interim statistical analyses involving hypothesis testing or any pre-specified stopping criteria for efficacy or futility on primary or secondary outcomes. Interim reports to the study team and data and safety monitoring board (DSMB) will consist of process measures such as protocol departures, missing values, missing forms, treatment regimen adherence, etc. and simple descriptive statistics on primary and safety outcomes of interest. In addition, weekly meetings with the study team will utilize central statistical monitoring techniques as a method of quality control and quality assurance for trial data on an ongoing basis. We foresee the DSMB requiring specific data listings or summarizations, but these will be specified at the time of the relevant DSMB meeting(s). At the request of the DSMB, we conducted an interim conditional power analysis, taking into consideration from both the QUARTET USA trial data as of August 2021 and further the QUARTET (Australia) results. These interim analyses, incorporating information to date, resulted in an updated recruitment target (and thus overall sample size goal). These interim analyses also resulted in the ultimate updates to the analytic strategy from an ANCOVA to one involving a mixed modeling approach to make use of both the six- and 12-week follow-up data for all participants. These modifications to the analytic plan resulted in an updated SAP (to version 2.0).

To preserve the integrity of the study, no formal final statistical analyses will occur until the REDCap database has been locked and all queries/discrepancies resolved; the date of database lock will be documented.

References:

1. Harris, P.A., et al., *Research electronic data capture (REDCap): a metadata-driven methodology and workflow process for providing translational reserach informatics support.* Journal of Biomedical Informatics, 2009. **42**(4): p. 377-381.
2. Scharfstein, D., et al., *Global sensitivity analysis for repeated measures studies with informative dropout: A fully parametric approach.* Statistics in Biopharmaceutical Research, 2014. **6**(4): p. 338-348.
3. Bahiru, E., et al., *Fixed-dose combination therapy for the prevention of atherosclerotic cardiovascular diseases.* Cochrane Database of Systematic Reviews, 2017(3).
